# Metformin sensitizes triple-negative breast cancer to histone deacetylase inhibitors by targeting FGFR4

**DOI:** 10.1186/s12929-025-01129-7

**Published:** 2025-03-17

**Authors:** Zhangyuan Gu, Fugui Ye, Hong Luo, Xiaoguang Li, Yue Gong, Shiqi Mao, Xiaoqing Jia, Xiangchen Han, Boyue Han, Yun Fu, Xiaolin Cheng, Jiejing Li, Zhiming Shao, Peizhen Wen, Xin Hu, Zhigang Zhuang

**Affiliations:** 1 Department of Breast Surgery, Shanghai Key Laboratory of Maternal-Fetal Medicine, Shanghai Institute of Maternal-Fetal Medicine and Gynecologic Oncology, Shanghai First Maternity and Infant Hospital, School of Medicine, Tongji University, No. 2699 West Gao-Ke Road, Shanghai, 201204 China; 2 Department of Breast Surgery, Key Laboratory of Breast Cancer in Shanghai, Fudan University Shanghai Cancer Center, No.688 Hong-Qu Road, Shanghai, 200032 China; 3 Department of Oncology, Shanghai Medical College, Fudan University, Shanghai, 200032 China; 4 Shanghai Henlius Biotech Inc., Shanghai, 200233 China; 5 Department of Medical Oncology, Shanghai Pulmonary Hospital, Cancer Institute, Tongji University School of Medicine, Shanghai, 200433 China; 6 Precision Cancer Medical Center, Affiliated to Fudan University Shanghai Cancer Center, No.688 Hong-Qu Road, Shanghai, 201315 China; 7 Department of General Surgery, School of Medicine, Organ Transplantation Clinical Medical Center of Xiamen University, Xiang’an Hospital of Xiamen University, Xiamen University, No. 2000 Xiang’an East Road, Xiamen, 361005 Fujian China; 8 Xiamen Human Organ Transplantation Quality Control Center, Xiamen Key Laboratory of Regeneration Medicine, Fujian Provincial Key Laboratory of Organ and Tissue Regeneration, School of Medicine, Organ Transplantation Institute of Xiamen University, Xiamen University, Xiamen, 361005 Fujian China

**Keywords:** Triple-negative breast neoplasms, Metformin, Histone deacetylase inhibitors, Drug synergism

## Abstract

**Background:**

Triple-negative breast cancer (TNBC) is characterized by high malignancy, strong invasiveness, and a propensity for distant metastasis, leading to poor prognosis and relatively limited treatment options. Metformin, as a first-line oral hypoglycemic agent, has garnered widespread research interest in recent years due to its potential in cancer prevention and treatment. However, its efficacy varies significantly across different tumor types. Histone deacetylase inhibitors (HDACi), such as SAHA, have demonstrated antitumor activity, but TNBC responds poorly to HDACi monotherapy, possibly due to feedback activation of the JAK-STAT pathway. Exploring the synergistic potential and underlying mechanisms of combining metformin with HDACi in TNBC treatment is crucial.

**Methods:**

We predicted the synergistic effects of metformin and SAHA in TNBC using multiple computational methods (CMap, DTsyn, and DrugComb). We also developed a cancer-specific compound mimic library (CDTSL) and applied a three-step strategy to identify genes fitting the "metformin sensitization" model. Subsequently, we evaluated the synergistic effects of metformin and SAHA in TNBC cell lines through cell proliferation, colony formation, and apoptosis assays. Furthermore, we investigated the molecular mechanisms of the combined treatment using techniques such as transcriptome sequencing, chromatin immunoprecipitation (ChIP), Western blotting, and measurement of extracellular acidification rate (ECAR). Additionally, we assessed the in vivo antitumor effects of the combined therapy in a nude mouse subcutaneous xenograft model.

**Results:**

CMap, DTsyn, and DrugComb all predicted the synergistic effects of SAHA and metformin in TNBC. The screening results revealed that HDAC10 played a key role in metformin sensitization. We found that the combination of metformin and SAHA exhibited synergistic antitumor effects (combination index CI < 0.9) in TNBC cell lines. Mechanistically, metformin inhibited histone acetylation on FGFR4, thereby blocking the feedback activation of FGFR4 downstream pathways induced by SAHA. Furthermore, metformin interfered with the glycolysis process induced by SAHA, altering the metabolic reprogramming of tumor cells. In in vivo experiments, the combined treatment of metformin and SAHA significantly inhibited the growth of subcutaneous tumors in nude mice.

**Conclusions:**

Metformin enhances the sensitivity of TNBC to HDAC inhibitors by blocking the FGFR4 pathway and interfering with metabolic reprogramming. When used in combination with SAHA, metformin exhibits synergistic antitumor effects. Our study provides a theoretical basis for the combined application of HDAC inhibitors and metformin, potentially offering a new strategy for the treatment of TNBC.

**Supplementary Information:**

The online version contains supplementary material available at 10.1186/s12929-025-01129-7.

## Introduction

Triple-Negative Breast Cancer (TNBC) is characterized by high malignancy, strong invasiveness, and a tendency for distant metastasis, leading to a generally poor prognosis [1, 2]. For early-stage TNBC patients, the standard treatment protocol is surgical resection combined with adjuvant chemotherapy/radiotherapy [3]. However, treatment options for advanced TNBC are relatively limited [4, 5].

In TNBC, the high expression of the histone deacetylase (HDAC) family is closely associated with the malignant phenotype of the tumor and poor prognosis. For instance, HDAC5 is linked to survival rates and metastasis [6], while HDAC2 and HDAC3 are highly expressed in invasive TNBC and correlate with low expression of estrogen receptor (ER), progesterone receptor (PR), and human epidermal growth factor receptor 2 (HER2) [7]. HDAC9 promotes tumor cell proliferation by inhibiting Bax and DR4 expression [8], and HDAC8 enhances proliferation by upregulating mutant P53 [9]. Additionally, HDAC1, HDAC6, and HDAC8 contribute to tumor invasion by increasing matrix metalloproteinase-9 (MMP-9) expression [10]. HDAC6 also deacetylates the mammalian sterile twenty kinase 1 (MST1), promoting cell proliferation [11]. Moreover, HDAC1, HDAC7, and HDAC8 are involved in maintaining the stemness of tumor stem cells, promoting TNBC proliferation and migration [12, 13]. These findings suggest that HDAC enzymes are critical players in TNBC progression and represent potential therapeutic targets [14].

Histone acetylation/deacetylation is an important epigenetic regulatory process. HDAC enzymes regulate gene transcription by deacetylating histones, influencing various biological processes such as cell cycle, apoptosis, and autophagy [15, 16]. HDACs are divided into four classes: Class I (HDAC1, 2, 3, 8), Class II (IIa: HDAC4, 5, 7, 9; IIb: HDAC6, 10), Class III (SIRT1-7), and Class IV (HDAC11) [15]. Currently, most HDAC inhibitors are pan-HDAC inhibitors, including Vorinostat (suberoylanilide hydroxamic acid, SAHA), Romidepsin, Belinostat, and Panobinostat, which have been approved by the U.S. Food and Drug Administration (FDA) for the treatment of hematological malignancies [17]. However, these drugs have limited efficacy in solid tumors and are prone to developing resistance [18]. Therefore, biomarker-guided stratified treatment strategies and combination therapies are needed to improve efficacy and delay the development of resistance [17].

SAHA, developed by Merck, is the world's first HDACi [19]. It has demonstrated antitumor activity in both hematologic and solid tumors, with patients generally tolerating its dosage well [20]. Although HDAC inhibitors are not yet FDA-approved for TNBC, they have shown efficacy in other breast cancer subtypes [21]. However, single-agent HDAC inhibitors have limited efficacy in TNBC, partly due to feedback activation of the JAK-STAT signaling pathway [22]. Therefore, combining HDAC inhibitors with other drugs for combination therapy may enhance efficacy and delay the onset of resistance.

Metformin, as a first-line oral hypoglycemic agent [23, 24], has been widely studied for its potential in cancer prevention and treatment [25–30]. It exerts anticancer effects by inhibiting mitochondrial oxidative phosphorylation and both AMPK-dependent and independent mechanisms [31–34]. However, its efficacy varies across cancer types [35, 36]. Drug repurposing, especially in combination with other therapies, has shown clinical benefits [37–39]. Thus, exploring the synergistic potential of metformin in TNBC combination therapies is crucial.

This study investigated the synergistic effects of SAHA and metformin in TNBC and their underlying mechanisms. Our findings provide a theoretical basis for the combined application of HDAC inhibitors and metformin, potentially offering a new strategy for the treatment of TNBC.

## Materials and methods

### Abbreviations

Given the extensive use of abbreviations in our study, We have compiled a comprehensive list of abbreviations and their corresponding full names in the in Supplementary Table S1

### Cell lines and compounds

We got the MDA-MB-231, BT-549, HCC1806, and Hs578T cell lines from the Shanghai Cell Bank Type Culture Collection Committee. These cell lines are often used as models for TNBC. The HEK293T cell line, derived from embryonic kidney cells, was also obtained from the same source. The American Type Culture Collection (ATCC) instructed us on how to culture these cells. The cells used in our experiments had been passaged no more than six times.

We purchased metformin (catalog No. D9351) from Beijing Solarbio Science & Technology Co., Ltd. and other small molecule compounds: JQ1 (catalog No. HY-13030), SAHA (catalog No. HY-10221) from MedChemexpress Co., Ltd. We diluted all compounds according to their manufacturers' instructions. Metformin was dissolved in fresh medium for cell tests and sterilized using a 0.22 μm membrane filter (Merck Millipore, catalog No. GVWP02500).

### Compound mimic library construction and lentivirus production

To construct the CRISPR/Cas9-based Drug Target Screening Library (CDTSL), the Cambridge Cancer Compound Library (CCCL), containing 247 compounds (Supplementary Table S2, Sheet 1) and 353 molecular targets, was derived from Selleck Chemicals (http://www.selleckchem.com). Six single-guide RNAs (sgRNAs) targeting each molecular target, along with 500 control sgRNAs, were designed using tools from the Genome Engineering website (http://www.genome-engineering.org/crispr/) (Supplementary Table S2, Sheet 2) and biosynthesized by SPECTRON (http://www.spectron.com.cn). Suzhou Hongxun Biotechnologies Co., Ltd. synthesized the sgRNA oligo pools and modified the sgRNA cloning vector (lentiCRISPR v2, Addgene catalog No. 52961) by replacing the antibiotic resistance label with a red fluorescent label to reduce false-positive rates during the screening process. The CDTSL was then generated by amplifying the sgRNAs and cloning them into the modified vector. Lentivirus production was performed following the protocol previously described by Dr. Feng Zhang [40].

### Two-step polymerase chain reaction (PCR) and MiSeq sequencing

Two-step PCR was used to amplify the barcode sequence for next generation sequence (NGS) using Illumina platform. Amplification was performed with 17 cycles for the first PCR and 15 cycles for the second PCR. For the first PCR, the amount of genomic DNA (gDNA) of each sample was calculated to ensure a 10000 × coverage of library. The assays were performed as previous reported [41].

### Three-step screening strategy for identifying metformin-sensitizing genes

To identify genes that fit the “metformin sensitization” model, we employed a three-step screening strategy using MAGeCK analysis. In the first step, we compared the Day 14 CDTSL group with the Day 0 group and screened genes that showed no significant effect (p > 0.05) when treated with CDTSL alone. In the second step, we compared the Day 14 CDTSL plus metformin group with the Day 0 group and identified genes that showed significant changes (p < 0.05) when treated with CDTSL plus metformin. In the third step, we compared the Day 14 CDTSL plus metformin group with the Day 14 CDTSL group to validate whether these genes showed significant differences (p < 0.05) between the CDTSL plus metformin and CDTSL alone treatments. Ultimately, we found that HDAC10 exhibited a significant metformin-sensitizing effect. Specifically, CRISPR knockout of HDAC10 showed borderline significance in the single treatment condition with CDTSL (p = 0.055780), suggesting that the loss of HDAC10 has a relatively mild impact on cell survival under these conditions. However, when combined with metformin treatment, HDAC10 knockout cells displayed a significant reduction in cell survival (CDTSL + Met vs. Day 0, p = 0.020368). Further comparison revealed that, compared to the single CRISPR screening condition, metformin treatment significantly enhanced the effect of HDAC10 knockout (CDTSL + Met vs. CDTSL, p = 0.022762). This progressive pattern of statistical significance strongly suggests that the loss of HDAC10 synergistically interacts with metformin, implying that HDAC10 may play a crucial role in the cellular response to metformin-induced metabolic stress (Supplementary Table S3).

### Differential analysis and enrichment analysis

Differential expression analysis was performed on the raw count matrix (Supplementary Table S4) using the DESeq2 package, with SAHA treatment group, metformin treatment group, and combined treatment group as experimental groups, and the corresponding untreated group or SAHA treatment group as control groups. The criteria for differentially expressed genes were adjusted according to different comparison combinations: p-value < 0.05 and |Log2FoldChange|> 1 for SAHA treatment group vs. control group, while p-value < 0.05 and |Log2FoldChange|> 0.8 for metformin treatment group vs. control group and combined treatment group vs. SAHA treatment group. Based on the screened differential genes, we conducted enrichment analysis using the clusterProfiler package [42] and calculated the zscore value for each enrichment entry using the GOplot package [43]. All visualizations were completed using the ggplot2 package in R. Additionally, we performed Gene Set Enrichment Analysis (GSEA) using the pre-defined All Canonical Pathways gene set from the MSigDB database [44, 45] to evaluate the distribution trend of genes in the gene list ranked by phenotype relevance, thereby assessing their contribution and relevance to the phenotype [46]. In the GSEA analysis, we applied the BH method for p-value correction, set the significance threshold to p.adj < 0.05 and FDR (qvalue) < 0.25, and set the number of calculations to 100,000 to ensure the reliability and stability of the results.

### Connectivity map drug sensitivity analysis (CMap)

To evaluate the potential synergistic effects of SAHA and metformin, we conducted CMap drug sensitivity analysis [47–49]. First, using the SAHA treatment group or the metformin treatment group as the experimental group, and the untreated group as the control group, we performed differential expression analysis on the raw count matrix (Supplementary Table S4-7) using the DESeq2 package. The criteria for differentially expressed genes were set to p-value < 0.05 and |Log2FoldChange|> 1. After screening, genes were sorted in descending order by Log2FoldChange value, and following CMap official validation standards, the top 150 up-regulated genes and top 150 down-regulated genes with the most significant changes in expression were selected as characteristic gene sets. Subsequently, we input these characteristic gene sets into the CLUE platform (https://clue.io/) for CMap query analysis. Query parameters were set as follows: L1000, Touchstone, individual query, and Latest. In the obtained CMap results, we further filtered with the conditions: pert_type as trt_cp (small molecule compound treatment) and cell line as breast cancer cell line MDA-MB-231. The significance threshold was set to fdr_q_nlog10 > 1.3 (equivalent to FDR < 0.05). Finally, we sorted the results in ascending order based on the Normalized Connectivity Score (norm cs), where a larger negative value indicates a higher correlation between the drug and the input gene characteristics, implying stronger potential sensitivity. To visually present the results, we selected the top 50 drugs and displayed them in a heat map format.

### Drug pair synergy prediction: DTsyn and DrugComb

We employed the Dual Transformer encoder model for drug pair Synergy prediction (DTSyn) model to predict the synergistic effects of drug combinations. DTSyn is a deep neural network model based on the multi-head attention mechanism, unique in its combination of two Transformer encoders of different granularities: a fine-granularity Transformer encoder for capturing chemical substructure-gene and gene–gene associations, and a coarse-granularity Transformer encoder for extracting chemical-chemical and chemical-cell line interactions [50]. We utilized the RNA-Seq expression profile data of cancer cell lines provided by the Cancer Cell Line Encyclopedia (CCLE) database (https://sites.broadinstitute.org/ccle/) as model input. The construction of the training set and the selection of drug pairs (METFORMIN and SAHA/VORINOSTAT) followed the Python script specifications provided by the PaddleHelix framework to ensure data consistency and reproducibility. After completing the DTSyn analysis, we used the ggplot2 package in R to generate bubble charts, visually presenting the Synergy Probability results. Drug combination sensitivity scores (CSS, S, ZIP, Bliss, HSA) were derived from the DrugComb database, and a combination of heat maps and bar charts was generated using the ggplot2 package in R.

### CERES score for pan-cancer cell line growth essentiality: revealing the critical degree of genes for cell survival

The CERES score is derived from the genome-wide CRISPR screening data published on the DepMap portal (https://depmap.org/portal/download/). This scoring system, based on the CERES algorithm, quantifies the necessity of approximately 17,000 candidate genes for cell survival [51]. The CERES algorithm employs alternating least squares regression, fitting the model to observed data through online computation to infer gene knockout effects and other related parameters. Subsequently, the gene knockout effects for each cell line are normalized, resulting in a score range with clear biological significance: a score of 0 indicates that the gene is non-essential for cell survival, while a score of 1 represents the median effect of common core essential genes, where gene knockout would lead to significant cell growth inhibition or death. The advantage of this scoring system lies in its ability to precisely quantify the importance of specific genes for the survival and proliferation of cell lines. Through CERES scores, researchers can quickly identify genes crucial for specific cancer types or subtypes.

### Expression profile and prognosis analysis: a systematic research approach integrating multi-source data

We obtained and organized RNAseq data from 33 tumor projects in the TCGA database (https://portal.gdc.cancer.gov) using the TCGAbiolinks package. These data were processed through the STAR pipeline and extracted in TPM (Transcripts Per Million) format. Among them, the TCGA-BRCA (Breast Invasive Carcinoma) project data received special attention. Prognosis-related clinical data were sourced from the study by Liu J et al. [52]. To ensure data quality, we excluded samples lacking clinical information and duplicates. All expression data underwent log2(value + 1) transformation. Expression profile analysis utilized the stats package and car package, employing the Mann–Whitney U test for statistical analysis, a non-parametric test method suitable for evaluating differences in gene expression levels. The survival package was used to test proportional hazards assumptions and fit survival regression models. To optimize grouping strategies, we applied the surv_cutpoint function from the survminer package to determine the optimal grouping cut-off value. Result visualization primarily relied on the survminer package and ggplot2 package. Conventional significance level markers were adopted: *p < 0.05; **p < 0.01; ***p < 0.001.

### Measurement of cell viability after drug treatment

To see how drugs interact with each other, we first set up concentration gradients of metformin in various 96-well plates. The concentration gradients of the compound were then set up in the horizontal wells of each 96-well plate. After an additional 72 h, we used the Cell Counting Kit-8 (Beyotime Biotechnology, catalog No. C0040) to determine cell viability. Finally, the combination index (CI) was determined using the Chou-Talalay approach to assess the interaction between the two drugs. The CI provides a quantitative measure of the interaction, indicating synergy (CI < 1), an additive effect (CI = 1), or antagonism (CI > 1) [53]. The experiments were repeated in triplicate.

To assess cell proliferation following transient overexpression or small interfering RNA (siRNA) transfection combined with drug treatment, we initially harvested the cells from culture dishes using trypsin on the first day. We then plated 5,000 cells per well for each treatment group in a 96-well plate. After 24 h, cell proliferation was evaluated using the Cell Counting Kit-8 (Beyotime Biotechnology, catalog No. C0040) according to the manufacturer's instructions. The experiments were conducted in triplicate.

### Colony formation assay

On the first day, 1000 MDA-MB-231 cells were seeded into each well of a six-well plate. After 24 h, different concentrations of SAHA (0, 0.125, 0.25, 0.5, or 1 μM), metformin (0, 2 or 4 mM), or their combinations were added to the wells. After 14 days, the formed colonies were stained using crystal violet and quantified using ImageJ software. The colony counts were then plotted using GraphPad Prism 7.0, with each experimental group performed in triplicate to ensure statistical reliability. Plots showing layered data were created as superplots [54].

### Measurement of cell apoptosis

We first digested the cells in the different treatment groups using trypsin without EDTA (Solarbio, catalog No. T1350). Then, we measured cell apoptosis according to the manufacturer's protocol for the Annexin V-FITC/Propidium Iodide (PI) Apoptosis Detection Kit (Yeasen Biotechnology, catalog No. 40302ES60). Cell apoptosis assays were conducted in triplicate.

### Chromatin immunoprecipitation (ChIP) assay

We provided rabbit monoclonal antibody anti-acetyl-histone H3K9 (1:50, Cell Signaling Technology, catalog No. 9649), anti-BRD4 (1:50, Cell Signaling Technology, catalog No. 13440) and Shanghai FuHeng Biotechnology Co., Ltd. accomplished ChIP assay. Each ChIP was performed with triplicate biological replicates. Plots showing layered data were created as superplots [54]. We list the primers used for the ChIP-qPCR assay in Supplementary Table S8.

### Quantitative real-time PCR (qPCR)

We digested cells in different treatment groups by trypsin from culture dishes, and we extracted total RNA according to the manufacturer's protocol of RNAeasy kit (Qiagen, 74104). Next, we reverse-transcribed total RNA into cDNA according to the manufacturer's protocol of HiScript III RT SuperMix for qPCR with a gDNA wiper (Vazyme Biotech Co.,Ltd., catalog No. R323-01). Then, we quantified qPCR analysis according to the manufacturer's protocol of ChamQ SYBR qPCR Master Mix (Vazyme Biotech Co.,Ltd., catalog No. Q311-02). We list the primers used for the qPCR assay in Supplementary Table S8. We used the comparative Ct (ddCt) method and normalized the values of target genes to the expression of β-actin (an endogenous control) [55].

### Western blot

We digested cells in different treatment groups by trypsin from culture dishes. After washing by PBS, 2% SDS was added, mixed, and placed in a 100 ℃ metal bath and reacted for 10–20 min. Next, we determined the protein concentrations according to the manufacturer’s protocol of a BCA protein assay kit (Vazyme Biotech Co.,Ltd., catalog No. E112-01). After adding 5 × SDS-PAGE sample buffer (Beyotime Biotech Inc., catalog No. P0015L), we boiled the proteins for another 5 min. Then, we separated proteins by SDS-PAGE and transferred the resolved proteins onto polyvinylidene difluoride membranes. After blocking the membranes for 60 min with 5% BSA in PBST, we blotted the membranes with the following primary rabbit monoclonal antibodies for 12–16 h at 4 ℃: anti-acetyl-α-Tubulin (Lys40) (1:1000, Cell Signaling Technology, catalog No. 5335), anti-FGFR4 (1:1000, Cell Signaling Technology, catalog No. 8562), anti-JAK1 (1:1000, Cell Signaling Technology, catalog No. 3344), anti-phospho-STAT3 (Tyr705) (1:1000, Cell Signaling Technology, catalog No. 9145), anti-STAT3 (1:1000, Cell Signaling Technology, catalog No. 4904), anti-cleaved-PARP (1:1000, Cell Signaling Technology, catalog No. 5625), anti-MCL-1 (1:1000, Cell Signaling Technology, catalog No. 5453), anti-phospho-Akt (Ser473) (1:1000, Cell Signaling Technology, catalog No. 4060), anti-Akt (1:1000, Cell Signaling Technology, catalog No. 4685), anti-phospho-Erk1/2 (Thr202/Tyr204) (1:1000, Cell Signaling Technology, catalog No. 4370), anti-Erk1/2 (1:1000, Cell Signaling Technology, catalog No. 9102). After extensive washing with PBST, the membranes were incubated for 2 h at room temperature with HRP-conjugated goat anti-rabbit antibody (1:5000, Affinity, catalog No. S0001), and we detected signals with an enhanced chemiluminescence substrate (Pierce Biotechnology). The image acquisition tool was Molecular Imager ChemiDoc XRS + (Bio-Rad Laboratories, Inc.) with Image Lab Software. Immunoblotting was carried out in three biological replicates. Plots showing layered data were created as superplots [54].

### siRNA transfection

All siRNAs were synthesized by Huzhou Hippo Biotechnology Co., Ltd. The siRNA sequences are listed in Supplementary Table S8. We first dissolved each freeze-dried powder siRNA in nuclease-free water to a final concentration of 20 μM. Next, we transiently transfected with siRNAs according to the manufacturer's protocol of Lipofectamine RNAiMAX Transfection Reagent (Thermo Fisher Scientific Inc., catalog No. 13778030). We removed the medium 6–12 h after the transfection and replaced it with a complete medium. Then, we performed other experiments after culturing for another 24–48 h.

### Transient overexpression

We purchased the empty control plasmid (pENTER) and overexpression plasmid (pENTER-FGFR4) from WZ Biosciences Inc. (Shandong, China). We verified the efficiency of overexpression by qPCR. We list primers used to construct these plasmids in Supplementary Table S8. According to the manufacturer's protocol of Lipofectamine 3000 Transfection Reagent (Thermo Fisher Scientific Inc., catalog No. L3000001), we gently mixed plasmid and Opti-MEM I (Invitrogen Inc., catalog No. 31985062) reduced serum medium and then incubated at room temperature for 5 min. Similarly, we mixed Lipo3000 and Opti-MEM I reduced serum medium and then incubated at room temperature for 5 min. Next, we gently mixed diluted plasmid and Lipo3000 and then incubated at room temperature for 20 min. Finally, we added the complex into the six-well plate, 2 mL per well. Then, we performed other experiments after culturing for another 24–48 h.

### Measurement of extracellular acidification rate (ECAR)

We digested cells in different treatment groups by trypsin from culture dishes. After counting by automated cell counter, they were reseeded into an XFe96 microplate by a multi-channel pipette at a density of 5000 cells per well in XF-Base Medium Minimal DMEM supplemented with glucose (25 mM) and pyruvate (1 mM). Each group had five replicate wells. After covering the lid, the XFe96 microplate was incubated overnight in a 37 °C, 5% CO2 humidified incubator. The day before we examined ECAR, we also warmed up the Seahorse XFe96 analyzer to room temperature and hydrated a probe plate overnight with an XF calibrant solution (200 μl per well) at 37 °C in a custom incubator without CO2. Next, we measured ECAR according to the manufacturer's protocol of a Seahorse XF Glycolysis Stress Test Kit (Agilent Technologies Inc., catalog No. 103020-100). The ECAR values were measured from 3 wells per sample, and the experiments were repeated three times.

### In vivo xenograft experiments

We purchased six-week-old nude female mice from Shanghai Jihui Laboratory Animal Co., Ltd. and treated MDA-MB-231 cells with Mycoplasma Elimination Reagent (Yeasen Biotechnology, catalog No. 40607ES03). Subcutaneous were established as previously described [56]. Each treatment group consisted of eight mice. We started oral drug treatment to mice since their tumors reached 100 mm^3^. We randomized mice into four groups: vehicle, SAHA (100 mg/kg), metformin (200 mg/kg), or the combination of SAHA (100 mg/kg) and metformin (200 mg/kg) as previously described [22, 57]. We dissolved metformin in saline and SAHA in a 50:50 mix of saline and PEG300 (MCE, catalog No. HY-Y0873). We treated mice with drugs for 28 continuous days. During this period, we measured the mice's weight and recorded the width and length of the tumors twice per week. We calculated the tumor volume using the formula: Tumor volume (V) = (length × width^2^)/2 as previously described [58]. Then we sacrificed these mice and fixed tumors in 4% paraformaldehyde (Absin Bioscience Inc., catalog No. abs9179).

### Immunohistochemistry (IHC)

We provided the following primary antibodies: rabbit monoclonal anti-FGFR4 (1:100, ABclonal Technology, catalog No. A9197), rabbit monoclonal anti-phospho-STAT3 (Tyr705) (1:100, ABclonal Technology, catalog No. AP0070), rabbit monoclonal anti-STAT3 (1:100, Abways Technology, catalog No. CY5016), rabbit polyclonal anti-MCL-1 (1:500, Servicebio, catalog No. GB11696) and Wuhan Servicebio Technology Co., Ltd. performed H&E and IHC staining of mice tumor sections. For immunohistochemistry, three fields of view per sample were imaged.

### Statistical analysis

We performed quantification and statistical analysis with GraphPad Prism 7.0. Data were presented as mean ± SD unless otherwise stated. Significance was determined by a two-tailed Student's t-test or one-way analysis of variance (ANOVA). Statistical significance thresholds were set at n.s. insignificant, *p < 0.05, **p < 0.01, ***p < 0.001, ****p < 0.0001.

## Results

### CMap analysis supported the synergistic effect of SAHA and metformin

**Fig. 1:**
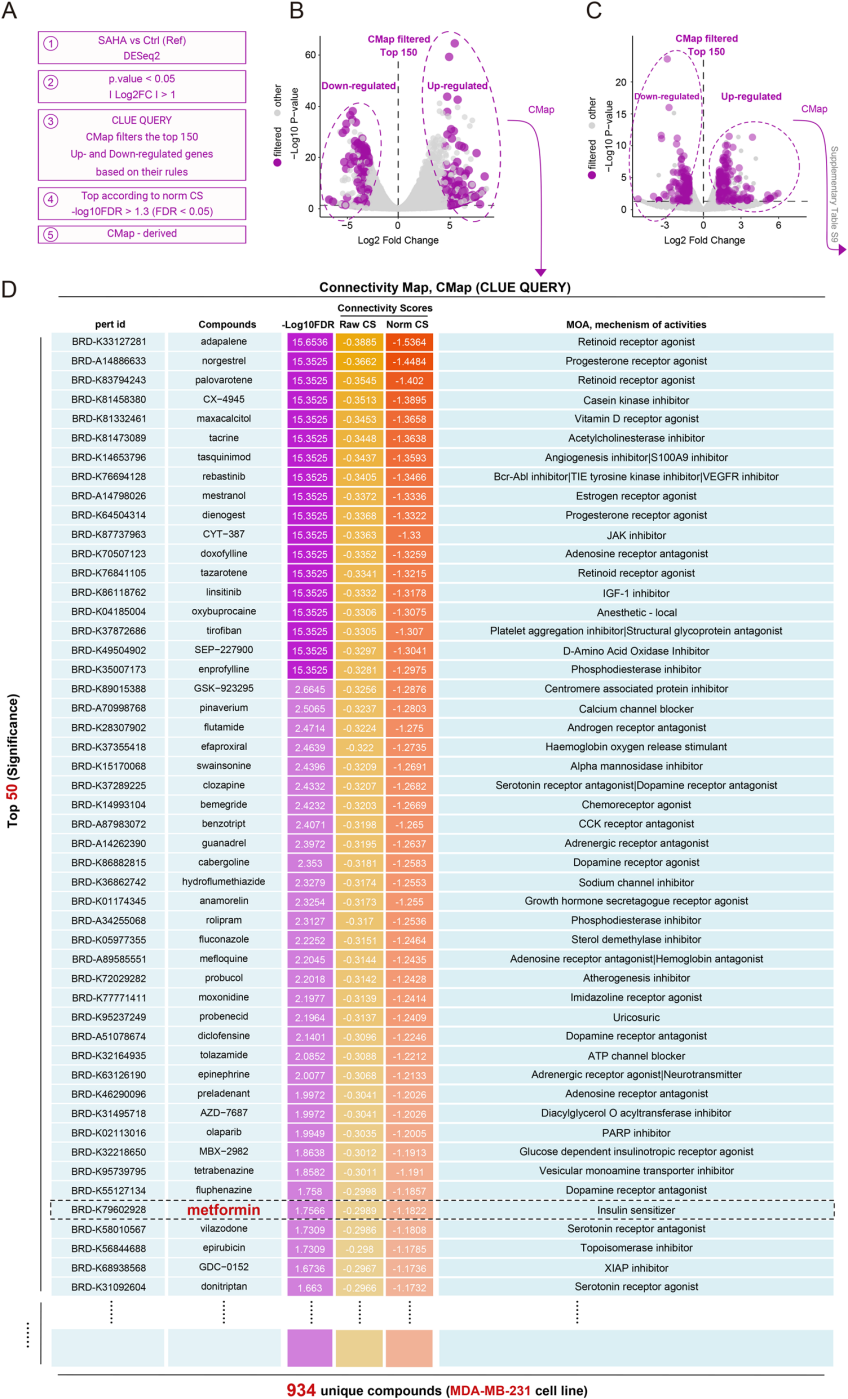
The overview of the CMap drug sensitivity analysis and its results were displayed. **A** Schematic representation of the CMap analysis workflow. **B**, **C** Volcano plots displaying differentially expressed genes. The highlighted genes corresponded to the top 150 upregulated and downregulated genes used as input for the CMap analysis. (**B**) compared the SAHA-treated group with the control group; (**C**) compared the Metformin-treated group with the control group. **D** Heatmap of the top 50 predicted sensitive drugs based on the gene expression profile of the SAHA-treated group, ranked by Normalized Connectivity Score, illustrating potential synergistic or antagonistic effects

Connectivity Map Drug Sensitivity Analysis (CMap) is a computational method based on gene expression data (Fig. 1A) used to reveal functional connections between drugs, diseases, and genes. This method is widely applied in drug repositioning, mechanism exploration, prediction of drug efficacy, and biomarker identification [47–49]. To investigate the potential synergistic effect of SAHA and metformin in triple-negative breast cancer (TNBC), we first analyzed the transcriptomic data of cells treated with each drug separately.

For the SAHA-treated cells, we generated a characteristic gene expression profile (Fig. 1B) and performed CMap analysis to evaluate whether metformin is a highly sensitive drug following SAHA treatment. The results showed that, under the restrictive breast cancer cell line condition (MDA-MB-231), we identified 934 drugs with statistically significant effects. These drugs were ranked in ascending order based on the Normalized Connectivity Score (norm_cs), where larger negative values indicated higher drug sensitivity. Metformin ranked relatively high among the 934 drugs (46/934) with a − Log10FDR of 1.7566 and Norm CS of − 1.1822 (Fig. 1D), suggesting a strong correlation with the gene expression pattern induced by SAHA treatment.

Similarly, we applied the same analysis process to the gene expression profile of metformin-treated cells (Fig. 1C) and performed CMap analysis. The results showed that SAHA also ranked statistically significant among all listed drugs, but not as highly as metformin did in the SAHA-treated cells (Supplementary Table S9).

### CDTSL identified a SAHA target enhancing metformin’s anticancer effect

Since library screening is an effective method for identifying synergistic drug targets, we further used metformin to screen for suitable targets for combination therapy in TNBC, thereby validating the synergistic effect between metformin and SAHA. Specifically, a mimic library creates drug-like effects through gene knockout instead of adding compounds. Knocking out the “X” gene mimics the action of an “X” inhibitor. By comparing changes in the target gene “X” under these conditions, we can identify genes and inhibitors that are ineffective alone but show enhanced anticancer effects when combined with metformin in TNBC cells (Supplementary Fig. S1A).

We constructed and screened CDTSL with the help of the Selleck Cambridge Cancer Compound Library. The Selleck Cambridge Cancer Compound Library is a unique collection of compounds targeting cancer. It encompasses most cancer targets, such as kinases, Notch signaling pathway, and metabolic targets. We collected 353 targets of the 247 compounds from the above-mentioned library. We then designed six sgRNA sequences for each target. We added 500 sgRNA sequences for the negative control. This work involved 2618 sgRNA sequences, which were the composition of the CDTSL sgRNA sequences (Supplementary Fig. S1B and Supplementary Table S2). After introducing CDTSL, metformin treatment, and library screening, we performed PCR amplification and NGS on different samples (Supplementary Fig. S1C).

To achieve our goal, we employed a three-step screening strategy to identify genes that fit the "metformin sensitization" model. In the first step, we screened genes that showed no significant effect when treated with CDTSL alone (p > 0.05), with a particular focus on the 185 genes with borderline significance (Supplementary Table S3, Sheet 1). In the second step, we identified genes that showed significant changes when treated with CDTSL plus metformin (p < 0.05) (Supplementary Table S3, Sheet 2). In the third step, we validated whether these genes showed significant differences between the CDTSL plus metformin and CDTSL alone treatments (p < 0.05) (Supplementary Table S3, Sheet 3). Ultimately, we identified 67 candidate genes that met the "metformin sensitization" criteria (Supplementary Fig. S1D and Supplementary Table S3, Sheet 4). Functional enrichment analysis revealed a significant enrichment of histone modification-related genes (Supplementary Fig. S1E).

Among these genes, SAHA’s target gene HDAC10, a key histone deacetylase, exhibited a significant metformin-sensitizing effect. HDAC10 is known to play a crucial role in cancer progression by regulating cell proliferation [59], apoptosis [60], migration and invasion [61], and angiogenesis [62]. Additionally, analysis of sequencing data from 1,462 breast cancer patients revealed a significant correlation between HDAC10 expression levels and patient prognosis (Supplementary Fig. S2). Therefore, the results of the library screening supported the synergistic effect of SAHA and metformin in TNBC.

### Metformin synergized with SAHA to inhibit TNBC growth

To further determine the appropriate cell lines for continued combination therapy experiments, we employed two additional computational methods to predict the effects of Metformin and SAHA (Vorinostat).

**Fig. 2:**
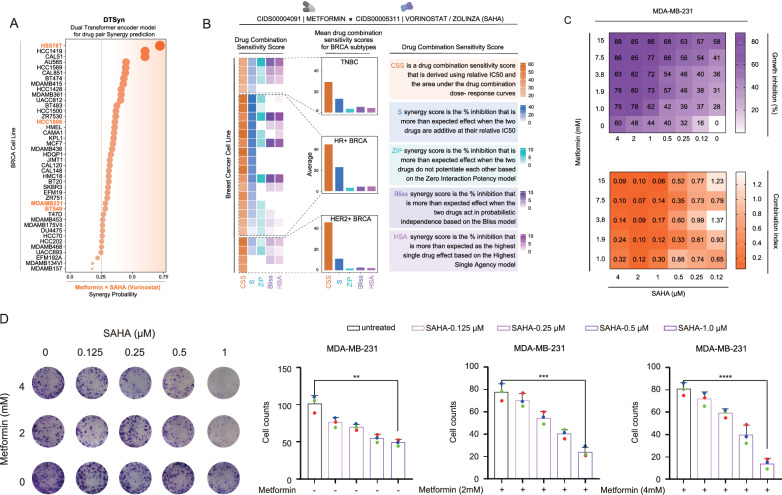
SAHA and metformin worked synergistically to inhibit TNBC. **A** The DTSyn algorithm calculated the drug synergy probability between Metformin and SAHA/Vorinostat in breast cancer cell lines. **B** Sensitivity scores for the drug combination (Metformin and Vorinostat/Zolinza [SAHA]) across different breast cancer subtypes. The heatmap on the left displayed the sensitivity of various breast cancer cell lines to the drug combination, the bar chart in the middle showed the average sensitivity score for each breast cancer subtype, and the definitions of the five sensitivity scores were provided on the right. **C** The percentage inhibition (up panel) and CI (down panel) at each concentration of the drugs were presented. MDA-MB-231 cells were treated with SAHA, metformin, or both at the concentrations as indicated. **D** The colony formation assay and its quantification of MDA-MB-231 cells treated with SAHA, metformin, or their combination were presented. MDA-MB-231 cells (1,000 per well) were seeded into six-well plates and treated after 24 h with varying concentrations of SAHA, metformin, or their combination. After 14 days, colonies were stained with crystal violet, quantified using ImageJ, and plotted with GraphPad Prism 7.0. All experiments were performed in triplicate. Error bars represented means ± SD from triplicates. *p < 0.05, **p < 0.01, ***p < 0.001, ****p < 0.0001

First, we used the DTSyn model, a deep neural network with multi-head attention, which effectively captures interactions between chemical substructures, genes, and cell lines. The model predicted a significant synergistic effect in most breast cancer cell lines, with the HS578T cell line showing a synergy probability close to 0.75 (Fig. 2A).

Next, we used the DrugComb database to assess the drug combination's synergy, focusing on the Combination Sensitivity Score (CSS) and Synergy Score (S). Both scores indicated strong synergy potential, particularly in breast cancer subtypes, despite other metrics like ZIP, Bliss, and HSA being lower (Fig. 2B).

We then conducted cell proliferation assays to determine if metformin synergizes with SAHA in inhibiting TNBC. We measured the inhibition rates at different drug concentrations and calculated the combination index (CI) for each. In MDA-MB-231 cells (Fig. 2C), the inhibition rate increased with higher concentrations of both SAHA and metformin. Most CIs were below 0.9, indicating a synergistic effect in inhibiting MDA-MB-231 cell growth. This synergy was also observed in Hs578T cells (Supplementary Fig. S3).

After assessing 72 h cell proliferation, we investigated the long-term effects of the drug combination using a colony formation assay. The results aligned with the short-term findings (Fig. 2D, Supplementary Fig. S4).

In summary, SAHA and metformin may induce similar drug-responsive gene expression patterns. These computational results not only align with our experimental observations, but also offer new research directions for exploring the molecular mechanisms underlying the combined use of SAHA and metformin.

### Metformin reversed SAHA-induced feedback activation by inhibiting histone acetylation on FGFR4

Previous study has shown that HDAC inhibitors increased the histone acetylation levels of the leukemia inhibitory factor receptor (LIFR) gene promoter region, recruiting the histone acetylation recognition protein-bromodomain-containing protein 4 (BRD4), which subsequently upregulated LIFR expression in tumor tissues. This elevated LIFR pathway activates the downstream JAK1-STAT3 signaling pathway, leading to treatment failure [22].

Additionally, research suggests that the feedback activation of the STAT3 signaling pathway may be the reason why TNBC, unlike other breast cancer subtypes, is not responsive to SAHA treatment. Therefore, inhibiting the STAT3 feedback activation induced by SAHA could be key to improving the efficacy of HDAC inhibitors in TNBC.

It is also known that metformin can target STAT3, inhibiting the proliferation of TNBC cells and inducing apoptosis [63].

Given that LIFR is just one of many membrane receptors capable of activating the JAK-STAT signaling pathway, we hypothesized that other receptors might also be feedback-activated by SAHA but inhibited by metformin. This could have partially explained why metformin reversed the insensitivity of TNBC cells to SAHA treatment.

**Fig. 3:**
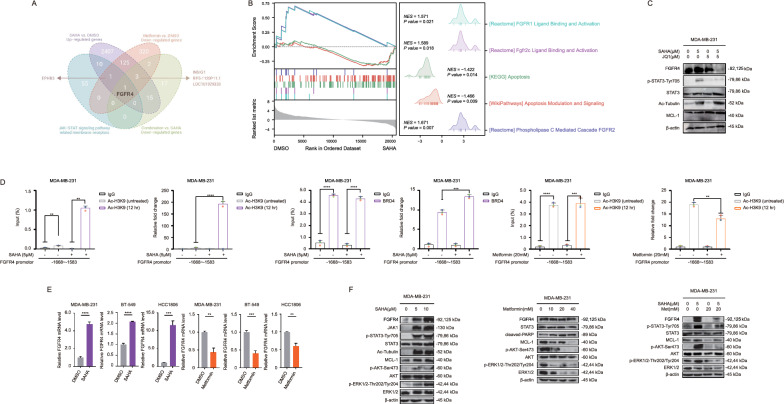
Metformin reversed SAHA-induced feedback activation by inhibiting histone acetylation on FGFR4. **A** The Venn diagram displayed the intersection of upregulated genes in the SAHA-treated group, downregulated genes in the Metformin-treated group, downregulated genes in the SAHA + Metformin combination group, and membrane receptor genes. **B** GSEA analysis was performed on the differential expression results between the SAHA-treated group and the control group. **C** Immunoblotting showed the change in FGFR4 and STAT3 phosphorylation. MDA-MB-231 cells pretreated with JQ1 for 24 h were exposed to SAHA for a further 12 h. FGFR4 and STAT3 phosphorylation changes were detected by immunoblotting. All bands were quantified from experiments repeated three times. **D** Histone acetylation on the FGFR4 promoter. (Left) MDA-MB-231 cells were treated with SAHA (5 μM) for 12 h before being subjected to ChIP assay using anti-acetylhistone H3K9 (Ac-H3K9) antibody followed by qPCR analysis using primers targeting the indicated FGFR4 promoter region. (Middle) BRD4 enrichment on the FGFR4 promoter. MDA-MB-231 cells were treated with SAHA (5 μM) for 12 h before being subjected to ChIP assay using anti-BRD4 antibody. qPCR analysis was performed using primers targeting the indicated FGFR4 promoter region. (Right) MDA-MB-231 cells were treated with Metformin (20 mM) for 48 h before being subjected to ChIP assay using anti-acetylhistone H3K9 (Ac-H3K9) antibody followed by qPCR analysis using primers targeting the indicated FGFR4 promoter region. All experiments were performed in triplicate. Error bars represent means ± SD from triplicates. *p < 0.05, **p < 0.01, ***p < 0.001, ****p < 0.0001. **E** FGFR4 mRNA level changes. MDA-MB-231, BT-549, and HCC1806 cells were treated with SAHA (5 μM) for 24 h or metformin (20 mM) for 48 h. Samples were analyzed by qPCR assay. Error bars represent means ± SD from triplicates. *p < 0.05, **p < 0.01, ***p < 0.001, ****p < 0.0001. **F** Immunoblotting showed the change in FGFR4 and STAT3 phosphorylation. (Left) MDA-MB-231 cells were treated with indicated SAHA (0, 5, 10 μM) for 12 h. (Middle) MDA-MB-231 cells were treated with indicated metformin (0, 10, 20, 40 mM) for 48 h. (Right) MDA-MB-231 cells pretreated with metformin (20 mM) for 36 h were exposed to SAHA (5 μM) for further hours. FGFR4 and STAT3 phosphorylation changes were detected by immunoblotting. All bands were quantified from experiments repeated three times

To investigate how metformin enhanced the effectiveness of SAHA in treating TNBC, we performed transcriptome sequencing to identify differentially expressed genes in the SAHA-treated (Supplementary Fig. S5A) and metformin-treated (Supplementary Fig. S5E) groups compared to controls (Supplementary Table S4). Gene Ontology and Kyoto Encyclopedia of Genes and Genomes (GO-KEGG) pathway enrichment analysis revealed that the SAHA-treated groups were enriched in pathways related to the response to fibroblast growth factor (FGFR) and the positive regulation of protein kinase activity (Supplementary Fig. S5A), with particular emphasis on FGFR-related pathways (Fig. 3B). Consistent with previous reports, the LIFR gene was significantly upregulated in the SAHA-treated groups compared to controls, as highlighted by the purple dot on the volcano plots (Supplementary Fig. S5D).

Next, we identified common elements among the upregulated genes in the SAHA-treated groups (Supplementary Fig. S5B), the downregulated genes in the metformin-treated groups (Supplementary Fig. S5F), and membrane receptors associated with the JAK-STAT signaling pathway (Supplementary Fig. S5B, C, F, G). We also overlapped the downregulated genes in the combination-treated groups (Fig. 3A) compared to the SAHA-treated groups, taking into account the functional nature of membrane receptors and their expected experimental effects. Fibroblast growth factor receptor 4 (FGFR4) emerged as a key intersecting gene (Fig. 3A, Supplementary Fig. S5D, H, Supplementary Table S10).

To test our hypothesis, we added the BRD4 inhibitor JQ1 to the SAHA treatment group and observed reduced FGFR4 protein expression (Fig. 3C, Supplementary Fig. S6A). ChIP experiments showed that SAHA increased histone acetylation on BRD4 and FGFR4 genes, while metformin inhibited it (Fig. 3D). We then confirmed that metformin disrupted SAHA-induced feedback activation of FGFR4 and its downstream pathways (FGFR4-JAK1-STAT3, FGFR4-AKT, FGFR4-ERK) at both the transcriptome (Fig. 3E) and protein levels (Fig. 3F, Supplementary Fig. S6B).

In addition, we evaluated cell apoptosis and proliferation after FGFR4 interference before SAHA treatment in MDA-MB-231 cells. We observed a notable increase in apoptosis and a marked inhibition of cell proliferation (Supplementary Fig. S7A, Fig. S8). Conversely, when FGFR4 was overexpressed using an expression plasmid in metformin-treated MDA-MB-231 cells, apoptosis was reduced, and cell proliferation was not inhibited (Supplementary Fig. S7C, Fig. S8). At the protein level, we also observed similar trends in the expression of the anti-apoptotic factor myeloid cell leukemia-1 (MCL-1) (Supplementary Fig. S7B, D).

In summary, metformin effectively reversed SAHA-induced feedback activation in TNBC by inhibiting histone acetylation on FGFR4, as demonstrated through histone modification (ChIP), transcriptome sequencing, and protein analysis. FGFR4 emerged as a key target in this process, confirming metformin's role in disrupting the feedback loop.

### Metformin enhanced SAHA’s efficacy in TNBC through metabolic reprogramming

**Fig. 4:**
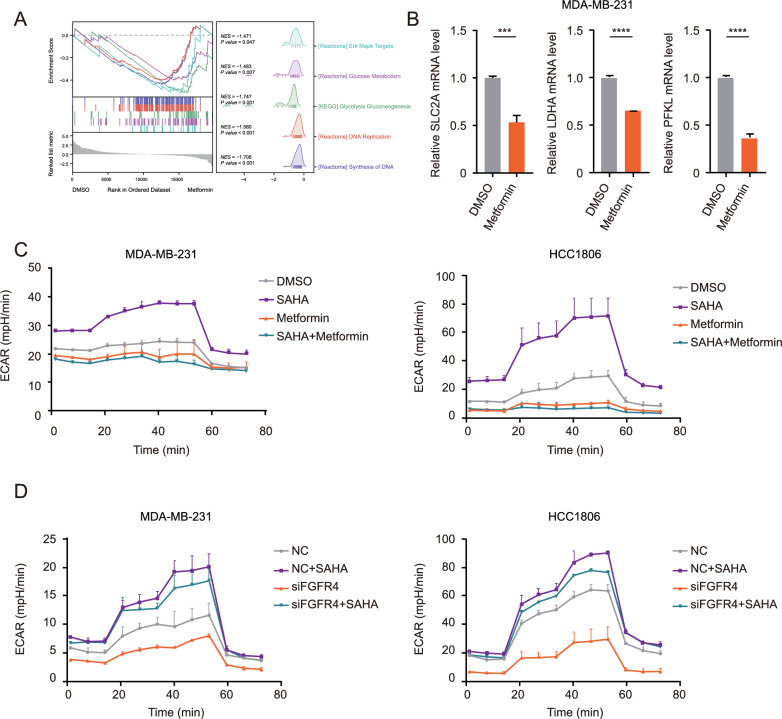
Metformin enhanced SAHA's efficacy in TNBC through metabolic reprogramming. **A** GSEA analysis was conducted on the differential expression results between the metformin-treated group and the control group. **B** The mRNA levels of metabolism-related genes (SLC2A, LDHA, PFKL) were measured. MDA-MB-231 cells were treated with 2 μM DMSO or 20 mM metformin for 48 h. **C** ECAR was measured in MDA-MB-231 and HCC1806 cells exposed to 2 μM DMSO, 5 μM SAHA, 20 mM metformin, or 5 μM SAHA + 20 mM metformin. The ECAR was analyzed using the Seahorse XF Glycolysis Stress Test Kit and the Seahorse XFe96 analyzer. The ECAR values were measured from 3 wells per sample, and the experiments were repeated three times. **D** ECAR was measured in MDA-MB-231 and HCC1806 cells transfected with NC or FGFR4 siRNAs for 48 h before exposure to 2 μM DMSO or 5 μM SAHA for 24 h. The ECAR was analyzed using the Seahorse XF Glycolysis Stress Test Kit and the Seahorse XFe96 analyzer. The ECAR values were measured from 3 wells per sample, and the experiments were repeated three times

To further explore how metformin inhibited the expression of FGFR4, through GO-KEGG pathway enrichment analysis, we found that the metformin treatment groups were enriched in pathways such as glycolysis/gluconeogenesis (Fig. 4A) compared with the control groups.

Given that metformin is a metabolic inhibitor [64], we hypothesized it might affect FGFR4 expression via metabolic pathways. Literature indicates that FGFR amplification in cancers is closely tied to glycolysis dependency [65]. We speculated that metformin might inhibit FGFR4 expression, thereby impacting glycolysis.

Our qPCR experiments showed that metformin suppresses several metabolic genes (e.g., SLC2A, LDHA, PFKL) (Fig. 4B). ECAR assays further revealed that metformin significantly reduced SAHA-induced glycolysis (Fig. 4C). Similarly, FGFR4 knockdown, even without metformin, also decreased SAHA-induced glycolysis (Fig. 4D).

This study revealed the key role and clinical significance of FGFR4 in pan-cancer and breast cancer through multidimensional analysis. CRISPR-Cas9 screening from the DepMap database showed that FGFR4 knockout significantly inhibited cell growth or caused cell death in various breast cancer cell lines (Supplementary Fig. S9A). FGFR4 exhibited differential expression across tumor types (Supplementary Fig. S9B) and was most highly expressed in PR-Negative, ER-Indeterminate, HER2-Positive, and PAM50 Her2 subtypes. Expression increased with advanced stage and was higher in patients under 60 (Supplementary Fig. S9C).

Survival analysis across four datasets consistently linked high FGFR4 expression with poorer prognosis, including shorter disease-specific, disease-free, and recurrence-free survival (Supplementary Fig. S9D–G). We also found that FGFR4 and related metabolic genes (SLC2A1, LDHA, PFKL) were upregulated in tumors and FGFR4 high-expression groups, suggesting FGFR4's role in tumor metabolism (Supplementary Fig. S9H-L).

In summary, this study highlighted the critical importance of FGFR4 in both pan-cancer and breast cancer.

### Combination of metformin and SAHA inhibited the growth of subcutaneous tumors in mice

**Fig. 5:**
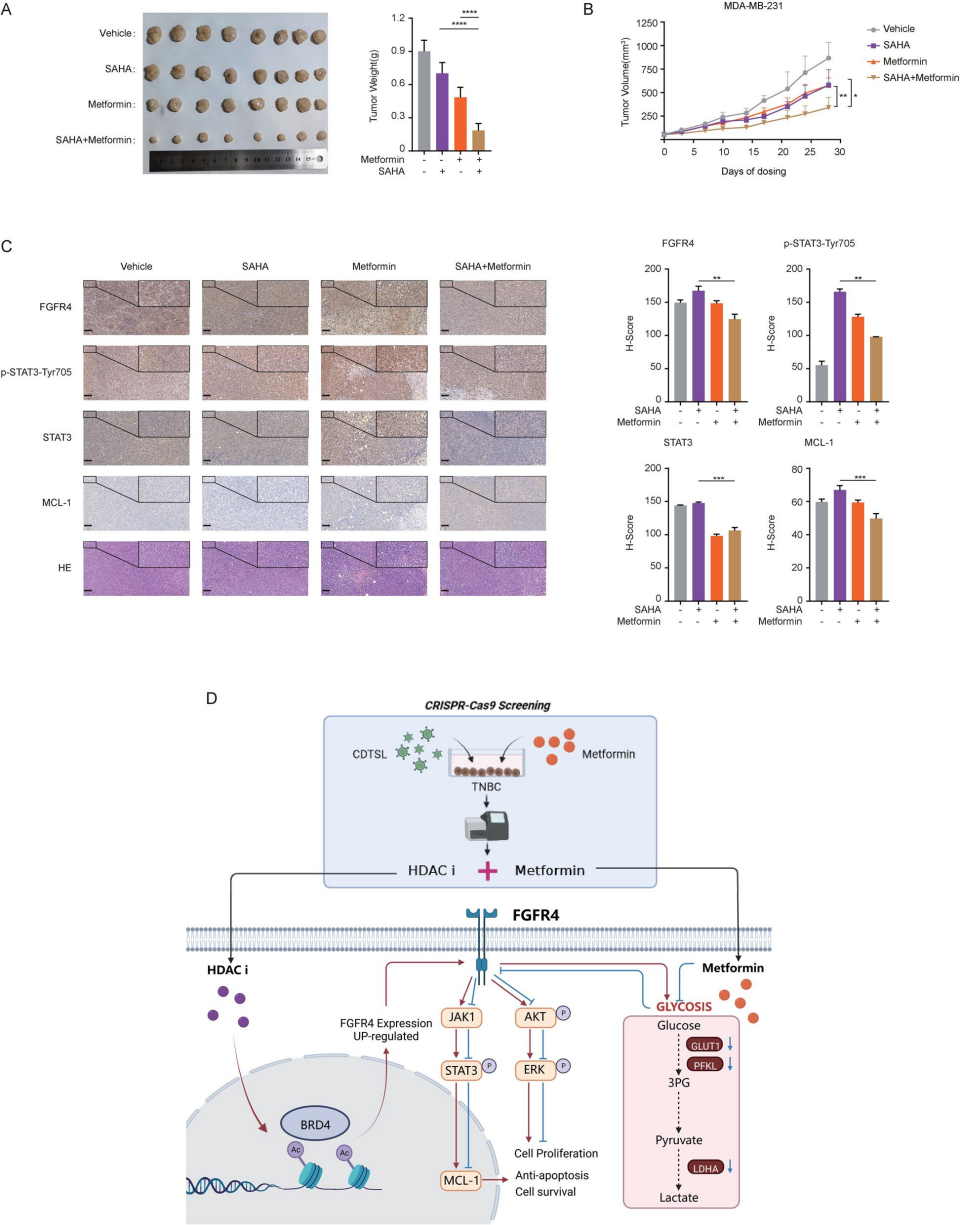
Combination of Metformin and SAHA inhibited the growth of subcutaneous tumors in mice. **A**, **B** Tumor images (A) and tumor growth curve (B) from each treatment group of the MDA-MB-231 xenograft model (n = 8). Mice were orally treated with SAHA (100 mg/kg) and metformin (200 mg/kg) alone or in combination daily for up to 4 weeks. Tumors were collected and measured 8 h after the last dosing (A) and the tumor growth curve was plotted by measuring the relative tumor volume twice per week (B). Scale bar, 1 cm. Error bars represented means ± SD from triplicates. *p < 0.05, **p < 0.01, ***p < 0.001, ****p < 0.0001. **C** Molecular alterations in the MDA-MB-231 subcutaneous xenograft model. Tumor samples, as described in (A), were collected 8 h after the last dosing, and intratumoral molecular changes were detected using immunohistochemistry analysis. Scale bar, 20 mm. **D** Proposed working model. We investigated the synergistic effects of the HDAC inhibitor SAHA and metformin in TNBC using multiple computational methods (CMap, DTsyn, and DrugComb) and bioinformatics predictions (CRISPR/Cas9 screening). The results were further validated through in vitro and in vivo experiments, elucidating the underlying mechanisms. Metformin inhibits the upregulation of histone acetylation on FGFR4, thereby suppressing the feedback activation induced by SAHA. This, in turn, affects its downstream pathways (FGFR4-JAK1-STAT3, FGFR4-AKT, and FGFR4-ERK), leading to the suppression of cancer cell proliferation and anti-apoptotic responses. Additionally, metformin influences glycolysis and metabolic genes (e.g., SLC2A, LDHA, PFKL), participating in metabolic reprogramming, which enhances the efficacy of SAHA in TNBC

Finally, we validated this through in vivo experiments and found that tumor growth in mice was significantly greater when the combination treatment was used compared to single-drug treatments (Fig. 5A, B). We quantified the changes in FGFR4 and its downstream IHC markers and reached consistent conclusions (Fig. 5C, Supplementary Fig. S10). Lastly, we presented a working model of our study to demonstrate the experimental mechanism (Fig. 5D). In our study, we found that metformin and the HDAC inhibitor SAHA work together in TNBC. Metformin inhibits the upregulation of histone acetylation on FGFR4, suppressing the feedback activation induced by SAHA. This disrupts its downstream pathways (FGFR4-JAK1-STAT3, FGFR4-AKT, and FGFR4-ERK), which helps inhibit cancer cell proliferation and resistance to apoptosis. Additionally, metformin impacts glycolysis and metabolic genes (e.g., SLC2A, LDHA, PFKL), contributing to metabolic reprogramming, which enhances the efficacy of SAHA in TNBC.

## Discussion

Metformin, primarily used for diabetes management, exhibits diverse applications in cancer treatment, particularly in TNBC [66–68]. In the research of combined therapy for TNBC, the mechanisms of action of metformin include enhancing the effectiveness of other anticancer therapies [69, 70], inhibiting tumor growth and metastasis [71–73], and inducing apoptosis [74]. The impact of metformin on cellular metabolic pathways, especially those related to glucose metabolism and mitochondrial function, is central to its anticancer effects [75, 76].

This study investigates the synergistic sensitizing effects of metformin and HDAC inhibitors in TNBC treatment. By employing multiple computational methods (CMap, DTsyn, and DrugComb) and bioinformatics approaches (CRISPR/Cas9 screening), we examined the combined effects of the HDAC inhibitor SAHA and metformin. The results were further validated through in vitro and in vivo experiments. On the one hand, HDAC inhibitors, by inhibiting histone deacetylase activity, increase the acetylation levels of histones in cells, restoring the expression of some tumor suppressor genes (such as p21 and p27), thereby inhibiting tumor growth [77]. On the other hand, HDAC inhibitors disrupt the balance between apoptosis and anti-apoptotic processes in cells, inducing the expression of pro-apoptotic genes, which then trigger tumor apoptosis through the mitochondrial (intrinsic pathway) or death receptor activation (extrinsic pathway) [78]. Although no HDAC inhibitor has been FDA-approved for TNBC treatment yet, promising clinical outcomes have been observed in other breast cancer subtypes [21]. Therefore, our study further elucidates how metformin enhances the anticancer effects of HDAC inhibitors in TNBC, providing a theoretical basis for the future application of HDAC inhibitors in TNBC.

The combination of metformin and HDAC inhibitors has shown synergistic effects in various cancers, such as osteosarcoma [79], bladder cancer [80], and cholangiocarcinoma [81]. For instance, metformin and TSA enhanced anti-tumor effects in osteosarcoma cells, independent of AMPK [79]. In bladder cancer, metformin activated AMPK and worked with Panobinostat to inhibit cell growth and apoptosis [80]. Our study uniquely explored the synergy of metformin and SAHA in TNBC, revealing that metformin enhanced SAHA's effect via FGFR4 feedback inhibition and by inhibiting glycolysis.

Our research demonstrates that the combination of metformin and SAHA shows significant synergistic sensitization in different TNBC cell lines (including MDA-MB-231 and Hs578T), indicating that synergistic effects are shared across other TNBC cell lines (Fig. 2C, Supplementary Fig. S3).

In seeking intersections between metformin and HDAC inhibitors, we focused mainly on the JAK-STAT pathway. According to the research conclusions of Geng et al. [22], screening different molecular subtypes of breast cancer cell lines for their sensitivity to SAHA revealed that TNBC cells were almost absent in the subsets most responsive to SAHA. In contrast, nearly all TNBC cells were in the subsets least responsive. It suggests that the feedback activation of the STAT3 signaling pathway is a crucial reason for the insensitivity of TNBC to SAHA treatment compared to other breast cancer subtypes.

We found that metformin not only downregulates the expression of FGFR4 but also alters the glycolysis pathway. Since metabolic reprogramming plays a crucial role in cancer development and progression, targeting cellular metabolism has become a hot topic in global anticancer drug research [82]. According to the research conclusions of Huang et al. [65], the overexpression of FGFR family is closely related to abnormal glucose metabolism in cancer. Therefore, we chose FGFR4 as our research subject to explore the metabolic reprogramming induced by FGFR4 activated by HDAC inhibitors in TNBC, thus sensitizing the response to metformin. Our study is the first to propose that the feedback activation of the FGFR4-JAK-STAT pathway may be one of the reasons for the poor efficacy of SAHA in TNBC. This phenomenon appears in TNBC rather than in other breast cancer subtypes. Our research also shows that this resistance can be reversed by metformin.

In this study, metformin demonstrates a stronger effect than FGFR4 knockdown in inhibiting SAHA-induced glycolysis (Fig. 4C, D). This difference may stem from metformin's direct regulation of metabolism via the AMPK pathway, which significantly suppresses glycolysis in cancer cells. In contrast, FGFR4 knockdown likely impacts glycolysis indirectly through the FGFR4-JAK-STAT pathway, resulting in a milder effect. Additionally, the role of FGFR4 may vary across different tumor types or cell contexts, which could explain why its impact on glycolysis inhibition is less pronounced than that of metformin.

Despite in vitro experiments demonstrating significant enhancement of anticancer effects by metformin, its translational value in clinical cancer treatment remains unclear. Recent prospective clinical trials targeting metformin for breast cancer treatment have not achieved significant results [83, 84], indicating that questions regarding the use of metformin alone or in combination and its applicability to specific breast cancer subtypes remain of significant research value. Our study focuses on the theme of ‘repurposing old drugs,’ specifically metformin. Although not the trendiest research topic, there is still vast potential for exploring how to enhance its anti-tumor efficacy.

In this study, by leveraging computational methods (CMap, DTsyn, and DrugComb) and bioinformatics predictions, we explored a combination treatment approach of HDAC inhibitors and metformin. We clarified how SAHA-induced FGFR4 activation prompts TNBC to undergo metabolic reprogramming, subsequently sensitizing TNBC to metformin. Ultimately, our research offers a potential treatment method for advanced breast cancer patients, particularly those with FGFR-activated TNBC.

## Conclusions

Our research demonstrates that metformin, in combination with the HDAC inhibitor SAHA, offers a novel and effective treatment strategy for triple-negative breast cancer (TNBC), showcasing significant advances in targeting this challenging cancer subtype through drug repurposing and mechanistic innovation.

## Supplementary Information

Supplementary Material 1: Fig. S1. Identification of Metformin-Sensitizing Genes via CRISPR-Cas9 Screening. A, The schematic diagram showed how CDTSL identified metformin-sensitizing genes and targeted inhibitors in TNBC. B, The schematic diagram showed the composition of the CDTSL sgRNA sequences. C, The schematic diagram showed the process of CDTSL library screening. D, The Venn diagram showed how 67 candidate genes were identified through MAGeCK analysis to meet the "metformin sensitization" model. E, Functional enrichment analysis revealed a significant enrichment of histone modification-related genes. Fig. S2. The sequencing results of 1462 breast cancer patients (5 cohorts) were displayed. The scatter plot in the upper-left corner compared the expression levels of HDAC10 in tumor tissues versus normal tissues. The remaining subplots analyzed survival differences between patients with high/low HDAC10 expression groups across different cohorts (GSE9893, GSE61304, GSE42568, GSE22219, and TCGA-BRCA) using Kaplan-Meier curves, covering endpoints such as overall survival (OS), disease-free survival (DFS), relapse-free survival (RFS), and progression-free survival (PFS). The p-values from the log-rank test were also annotated. Fig. S3. Combination Efficacy of SAHA and Metformin in TNBC Cell Lines. A, The IC50 curves for SAHA (purple curve) and metformin (orange curve) were shown. The left panel displayed the percentage inhibition (%), while the right panel presented the combination index (CI) at each drug concentration. MDA-MB-231 cells were treated with SAHA, metformin, or both at the indicated concentrations. B, The IC50 curves for SAHA (purple curve) and metformin (orange curve) were shown. The left panel displayed the percentage inhibition (%), while the right panel presented the combination index (CI) at each drug concentration. Hs578T cells were treated with SAHA, metformin, or both at the indicated concentrations. Fig. S4. Colony formation and quantification of MDA-MB-231 cells treated with SAHA, metformin, and combinations. The formed colonies and quantification of MDA-MB-231 cells treated with different concentrations of SAHA (0, 0.125, 0.25, 0.5, or 1 μM), metformin (0, 2 or 4 mM), or their combinations were displayed (in three replicates). Fig. S5. We analyzed differential gene expression, enrichment, and membrane receptor intersections for SAHA and metformin treatments. A, GO-KEGG enrichment analysis of differentially expressed genes (p-value < 0.05 and |Log2FoldChange| > 1) between the SAHA-treated group and the control group. B, Venn diagram showing the intersection of differentially expressed genes between the SAHA-treated group and the control group with membrane receptor genes. C, Heatmap displaying the intersection of differentially expressed genes between the SAHA-treated group and the control group with membrane receptor genes. D, Volcano plot of the differential expression analysis between the SAHA-treated group and the control group (p-value < 0.05 and |Log2FoldChange| > 1). E, GO-KEGG enrichment analysis of differentially expressed genes (p-value < 0.05 and |Log2FoldChange| > 0.8) between the Metformin-treated group and the control group. F, Venn diagram showing the intersection of differentially expressed genes between the Metformin-treated group and the control group with membrane receptor genes. G, Heatmap displaying the intersection of differentially expressed genes between the Metformin-treated group and the control group with membrane receptor genes. H, Volcano plot of the differential expression analysis between the Metformin-treated group and the control group (p-value < 0.05 and |Log2FoldChange| > 0.8). Fig. S6. Protein quantification data from Fig. 3. A, Protein quantification from Fig. 3C showing changes in FGFR4 and STAT3 phosphorylation. MDA-MB-231 cells pretreated with JQ1 for 24 hours were exposed to SAHA for an additional 12 hours. FGFR4 and STAT3 phosphorylation changes were detected by immunoblotting. All bands were quantified from experiments repeated three times. B, Protein quantification from Fig. 3F showing changes in FGFR4 and STAT3 phosphorylation. (Left) MDA-MB-231 cells were treated with the indicated concentrations of SAHA (0, 5, 10 μM) for 12 hours. (Middle) MDA-MB-231 cells were treated with the indicated concentrations of metformin (0, 10, 20, 40 mM) for 48 hours. (Right) MDA-MB-231 cells pretreated with metformin (20 mM) for 36 hours were exposed to SAHA (5 μM) for an additional 12 hours. FGFR4 and STAT3 phosphorylation changes were detected by immunoblotting. All bands were quantified from experiments repeated three times. Fig. S7. We introduced FGFR4 siRNA with SAHA and overexpressed FGFR4 with metformin to observe apoptosis, proliferation, and protein changes. A, Cell apoptosis and growth assays. MDA-MB-231 cells were transfected with non-targeting control (NC) or FGFR4 siRNAs for 48 hours, followed by SAHA treatment for an additional 24 hours for apoptosis analysis, and 72 hours for cell growth analysis. B, Immunoblotting and protein quantification of FGFR4 and MCL-1 change. MDA-MB-231 cells pretreated with non-targeting control (NC) or FGFR4 siRNAs for 48 hours, followed by SAHA (5μM) treatment for an additional 24 hours for Immunoblotting. FGFR4 and MCL-1 change was detected by immunoblotting. All the bands were quantified from experiments repeated three times. C, Cell apoptosis and growth assays. MDA-MB-231 cells were transfected with control or FGFR4 plasmid for 48 hours, followed by metformin treatment for an additional 48 hours for apoptosis analysis, and 72 hours for cell growth analysis. D, Immunoblotting and protein quantification of FGFR4 and MCL-1 change. MDA-MB-231 cells pretreated with control or FGFR4 plasmid for 48h, followed by metformin (20mM) treatment for an additional 48 hours for Immunoblotting. FGFR4 and MCL-1 change was detected by immunoblotting. All the bands were quantified from experiments repeated three times. Fig. S8. Cell apoptosis analysis shown in Fig. S7, performed in triplicates. Fig. S9. Multidimensional analysis of FGFR4 gene necessity, expression profiles, and prognostic impact in pan-cancer and breast cancer. A, DepMap database CRISPR-Cas9 whole-genome screening results: Displaying the top 200 pan-cancer cell lines ranked by FGFR4 CERES scores, reflecting the importance of FGFR4 for cell survival. B, FGFR4 expression levels across various cancer types: Comparison between tumor and normal tissues. C, Association analysis between FGFR4 expression and various clinical features: Including PR status, ER status, HER2 status, PAM50, Pathologic T stage, Pathologic N stage, Pathologic M stage, Pathologic stage, Age, Race, Menopause status, and Histological type. D, Correlation between FGFR4 expression levels and disease-specific survival (DSS) in breast cancer patients (TCGA-BRCA dataset). E, Relationship between FGFR4 expression levels and disease-free survival (DFS) in breast cancer patients (GSE21653 dataset). F, Association between FGFR4 expression levels and relapse-free survival (RFS) in breast cancer patients (GSE9893 dataset). G, Correlation between FGFR4 expression levels and relapse-free survival (RFS) in breast cancer patients (GSE22219 dataset). H-J, Comparison of FGFR4, SLC2A1, and LDHA gene expression between cancer and normal tissues. K, Differential expression of PFKL gene between high and low FGFR4 expression groups. L, Differential expression of SLC2A1 gene between high and low FGFR4 expression groups. Fig. S10. We quantified intratumoral molecular changes using randomly selected visual fields and immunohistochemical metrics. Three visual fields were randomly selected from each group after immunohistochemistry, and the molecular changes shown in Fig. 5 were quantified using two immunohistochemical metrics.

Supplementary Material 2: Table S1. Acronym and Full Name. Table S2. SgRNA Sequences of Genes in the CDTSL Library. Table S3. MAGeCK negative P-values for Day 14 CDTSL vs. Day 0, Day 14 CDTSL(+Met) vs. Day 0, Day 14 CDTSL(+Met) vs. Day 14 CDTSL, and the list of 67 metformin-sensitizing genes identified through the three-step screening strategy. Table S4. RNA-seq counts for SAHA vs Ctrl, Metformin vs Ctrl, and Combination vs SAHA. Table S5. Differential analysis results of SAHA vs Control. Table S6. Differential analysis results of Metformin vs Control. Table S7. Differential analysis results of SAHA combined with Metformin vs SAHA alone. Table S8. List of Primers Used for ChIP-qPCR, qPCR, Overexpression, and siRNA Experiments. Table S9. CMap analysis of the gene expression profile in metformin-treated cells assessed whether SAHA (Vorinostat) is a highly sensitive drug after metformin treatment. Table S10. 129 intersection genes between upregulated genes after SAHA treatment and downregulated genes after Metformin treatment.

## Data Availability

The library screening data of CDTSL were uploaded to the NCBI Sequence Read Archive (SRA) under BioProject IDs: PRJNA1119744, PRJNA1119748, and PRJNA1119749. The RNA-seq results are provided in Supplementary Table S4. The authors confirm the availability of data and materials.
