# Supplementary figures and images for: Metformin sensitizes triple-negative breast cancer to histone deacetylase inhibitors by targeting FGFR4
Source: J Biomed Sci. 2025 Mar 17;32:36. doi: 10.1186/s12929-025-01129-7 (PMC11912690; doi:10.1186/s12929-025-01129-7)

A

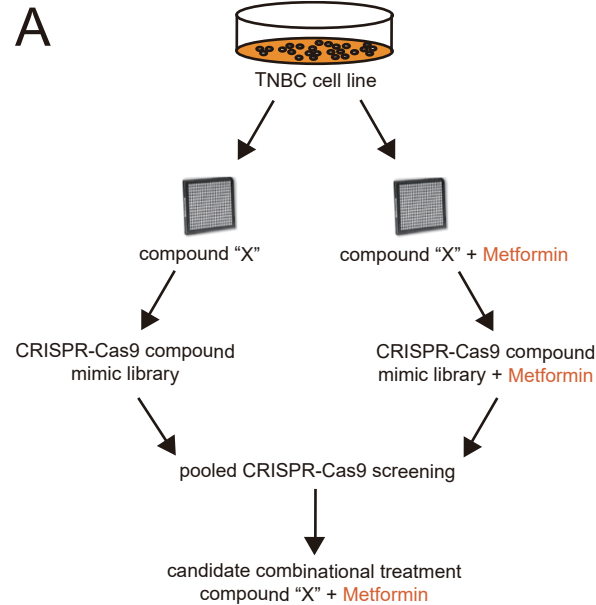

B

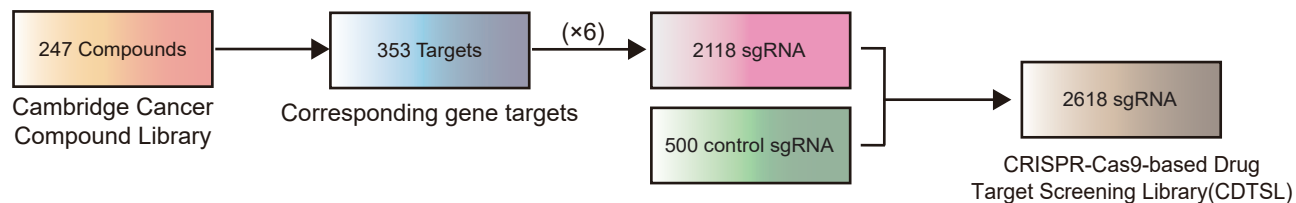

C

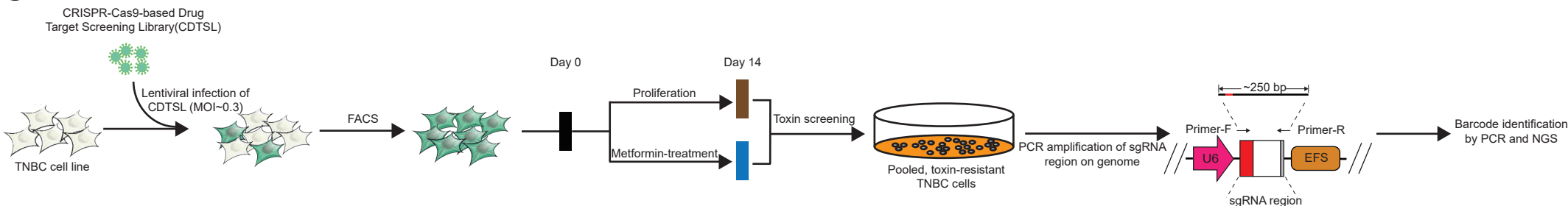

D

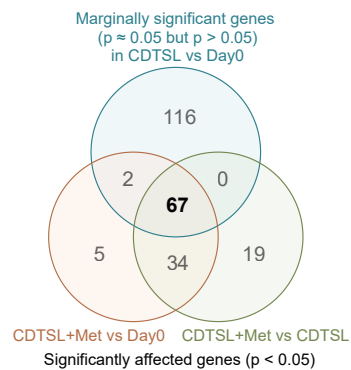

E

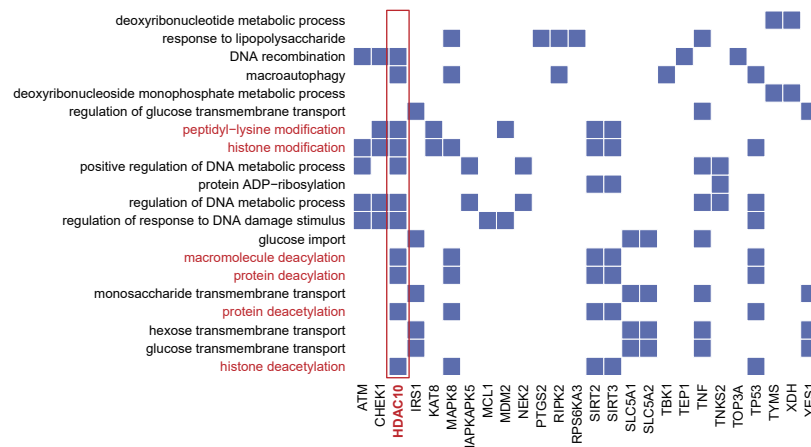

Supplement: Supplementary file 1 — Supplementary Material 1: Fig. S1. Identification of Metformin-Sensitizing Genes via CRISPR-Cas9 Screening. A, The schematic diagram showed how CDTSL identified metformin-sensitizing genes and targeted inhibitors in TNBC. B, The schematic diagram showed the composition of the CDTSL sgRNA sequences. C, The schematic diagram showed the process of CDTSL library screening. D, The Venn diagram showed how 67 candidate genes were identified through MAGeCK analysis to meet the "metformin sensitization" model. E, Functional enrichment analysis revealed a significant enrichment of histone modification-related genes. Fig. S2. The sequencing results of 1462 breast cancer patients (5 cohorts) were displayed. The scatter plot in the upper-left corner compared the expression levels of HDAC10 in tumor tissues versus normal tissues. The remaining subplots analyzed survival differences between patients with high/low HDAC10 expression groups across different cohorts (GSE9893, GSE61304, GSE42568, GSE22219, and TCGA-BRCA) using Kaplan-Meier curves, covering endpoints such as overall survival (OS), disease-free survival (DFS), relapse-free survival (RFS), and progression-free survival (PFS). The p-values from the log-rank test were also annotated. Fig. S3. Combination Efficacy of SAHA and Metformin in TNBC Cell Lines. A, The IC50 curves for SAHA (purple curve) and metformin (orange curve) were shown. The left panel displayed the percentage inhibition (%), while the right panel presented the combination index (CI) at each drug concentration. MDA-MB-231 cells were treated with SAHA, metformin, or both at the indicated concentrations. B, The IC50 curves for SAHA (purple curve) and metformin (orange curve) were shown. The left panel displayed the percentage inhibition (%), while the right panel presented the combination index (CI) at each drug concentration. Hs578T cells were treated with SAHA, metformin, or both at the indicated concentrations. Fig. S4. Colony formation and quantification o [file 12929_2025_1129_MOESM1_ESM.zip › Fig. S1.pdf]

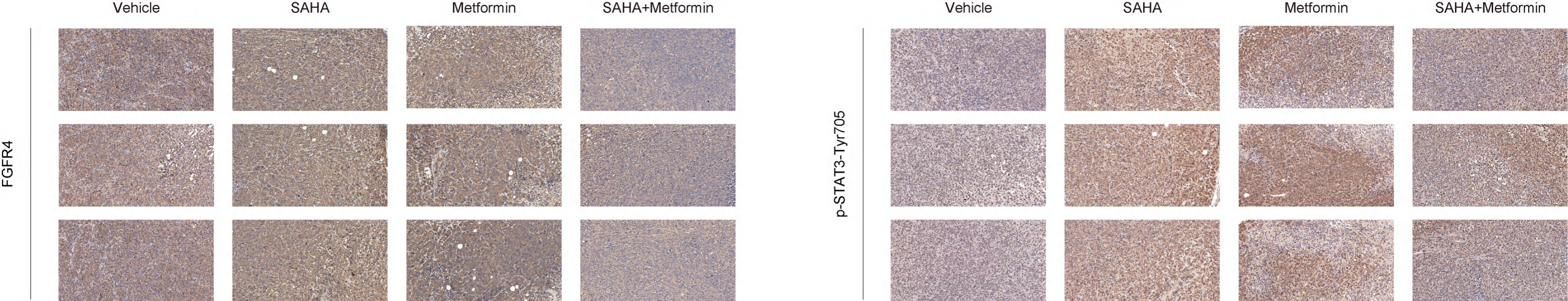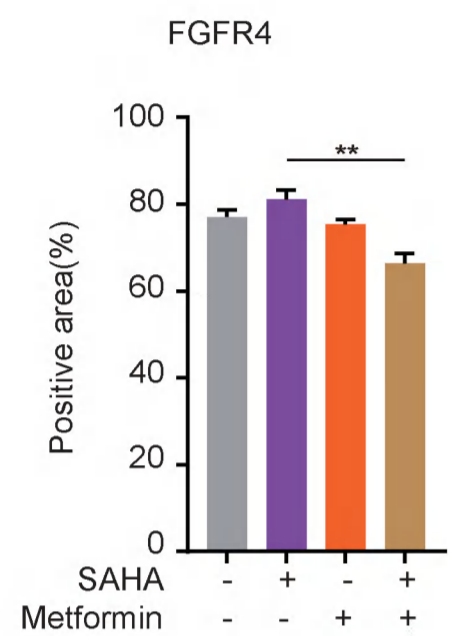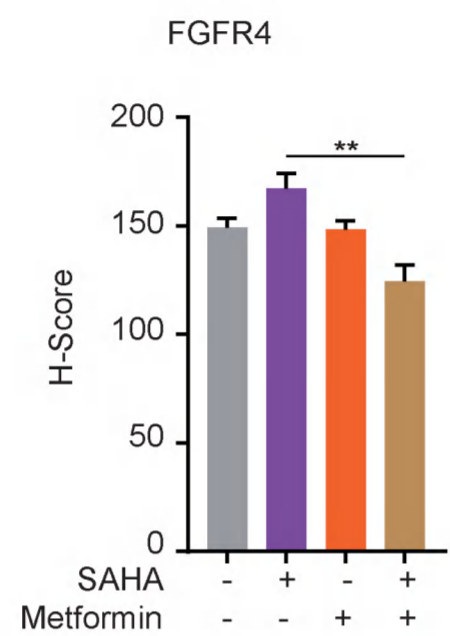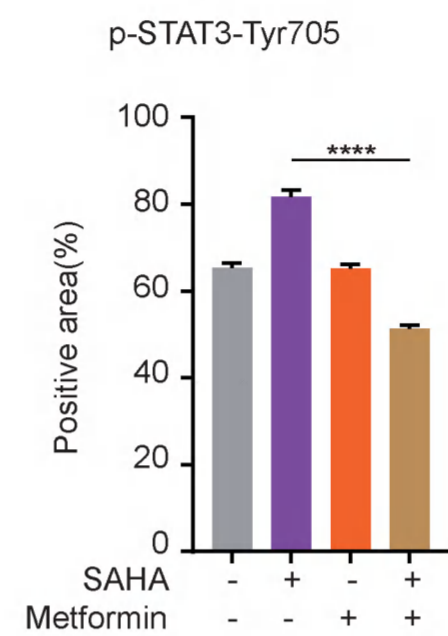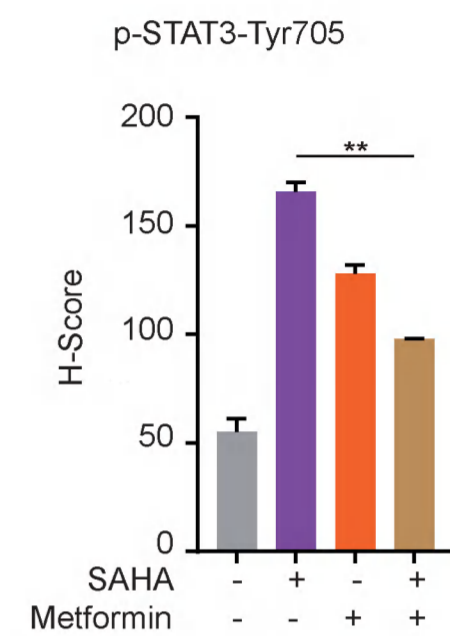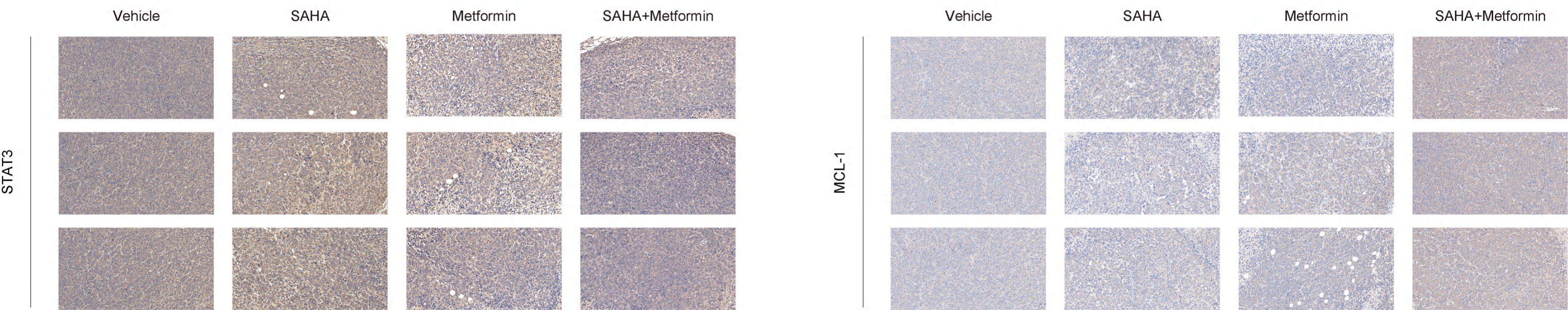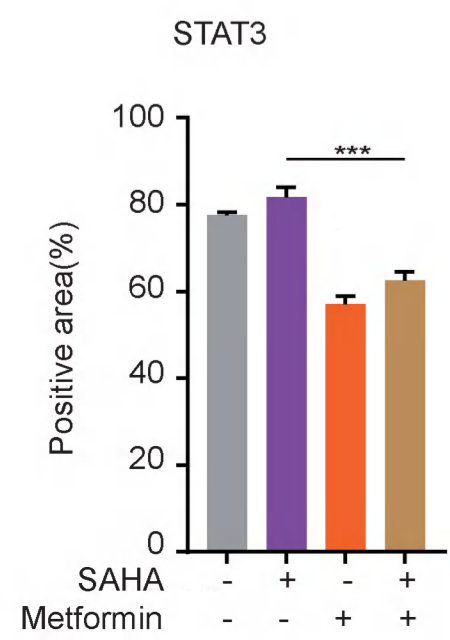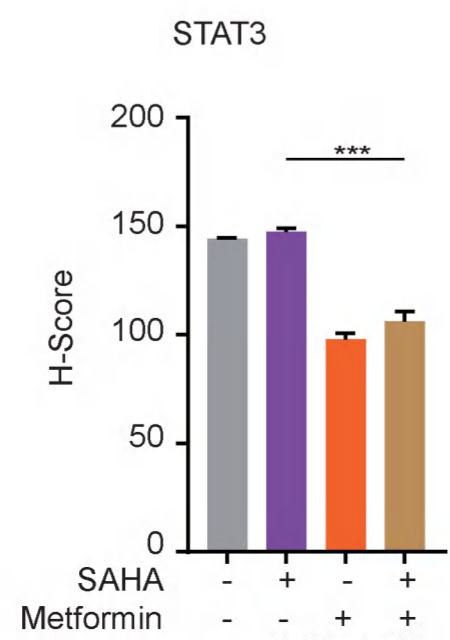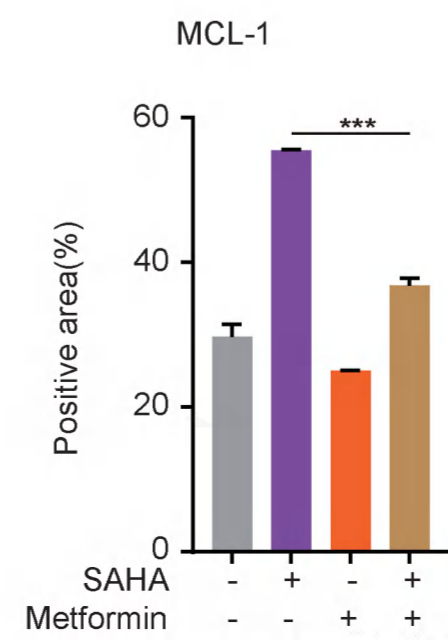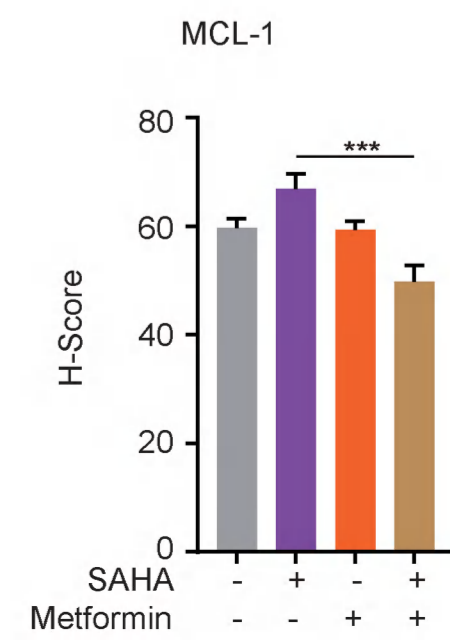

Supplement: Supplementary file 1 — Supplementary Material 1: Fig. S1. Identification of Metformin-Sensitizing Genes via CRISPR-Cas9 Screening. A, The schematic diagram showed how CDTSL identified metformin-sensitizing genes and targeted inhibitors in TNBC. B, The schematic diagram showed the composition of the CDTSL sgRNA sequences. C, The schematic diagram showed the process of CDTSL library screening. D, The Venn diagram showed how 67 candidate genes were identified through MAGeCK analysis to meet the "metformin sensitization" model. E, Functional enrichment analysis revealed a significant enrichment of histone modification-related genes. Fig. S2. The sequencing results of 1462 breast cancer patients (5 cohorts) were displayed. The scatter plot in the upper-left corner compared the expression levels of HDAC10 in tumor tissues versus normal tissues. The remaining subplots analyzed survival differences between patients with high/low HDAC10 expression groups across different cohorts (GSE9893, GSE61304, GSE42568, GSE22219, and TCGA-BRCA) using Kaplan-Meier curves, covering endpoints such as overall survival (OS), disease-free survival (DFS), relapse-free survival (RFS), and progression-free survival (PFS). The p-values from the log-rank test were also annotated. Fig. S3. Combination Efficacy of SAHA and Metformin in TNBC Cell Lines. A, The IC50 curves for SAHA (purple curve) and metformin (orange curve) were shown. The left panel displayed the percentage inhibition (%), while the right panel presented the combination index (CI) at each drug concentration. MDA-MB-231 cells were treated with SAHA, metformin, or both at the indicated concentrations. B, The IC50 curves for SAHA (purple curve) and metformin (orange curve) were shown. The left panel displayed the percentage inhibition (%), while the right panel presented the combination index (CI) at each drug concentration. Hs578T cells were treated with SAHA, metformin, or both at the indicated concentrations. Fig. S4. Colony formation and quantification o [file 12929_2025_1129_MOESM1_ESM.zip › Fig. S10.pdf]

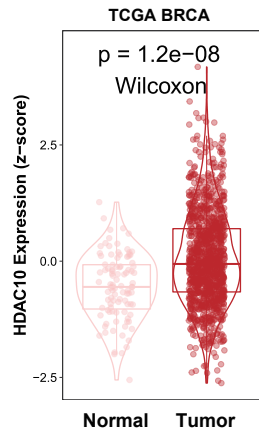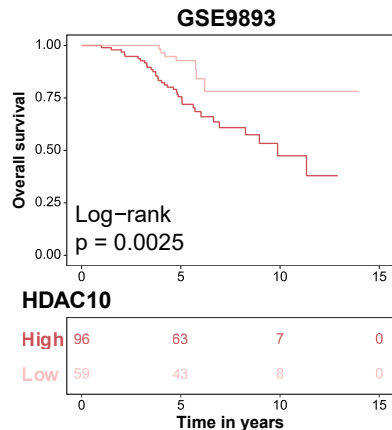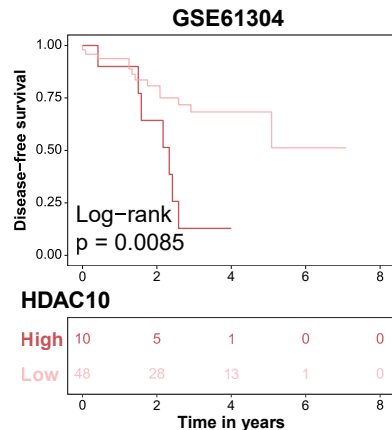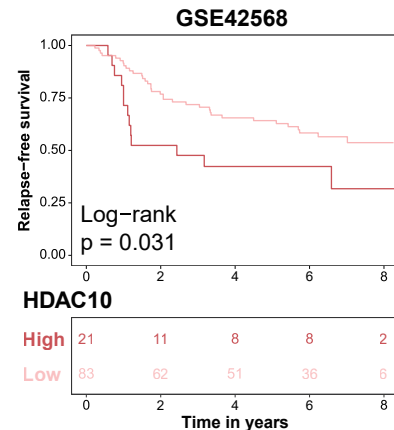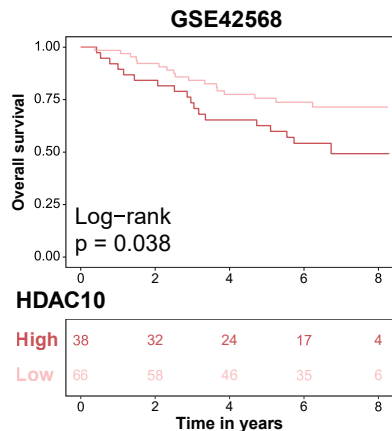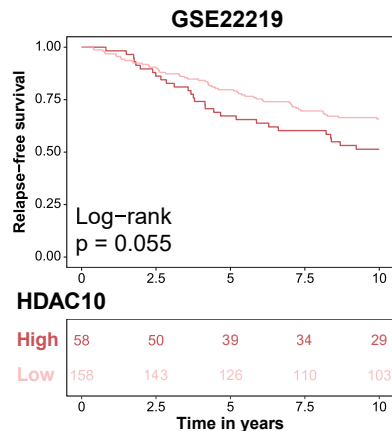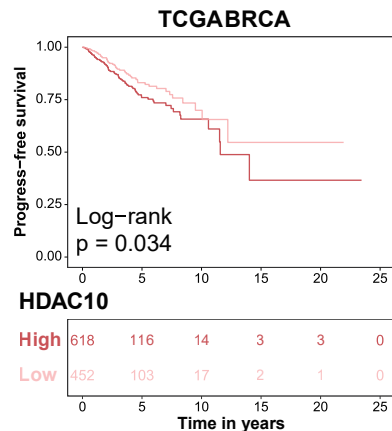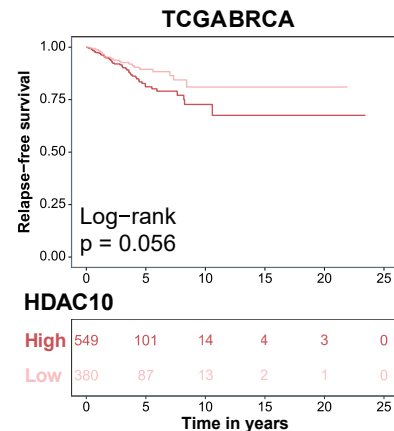

Supplement: Supplementary file 1 — Supplementary Material 1: Fig. S1. Identification of Metformin-Sensitizing Genes via CRISPR-Cas9 Screening. A, The schematic diagram showed how CDTSL identified metformin-sensitizing genes and targeted inhibitors in TNBC. B, The schematic diagram showed the composition of the CDTSL sgRNA sequences. C, The schematic diagram showed the process of CDTSL library screening. D, The Venn diagram showed how 67 candidate genes were identified through MAGeCK analysis to meet the "metformin sensitization" model. E, Functional enrichment analysis revealed a significant enrichment of histone modification-related genes. Fig. S2. The sequencing results of 1462 breast cancer patients (5 cohorts) were displayed. The scatter plot in the upper-left corner compared the expression levels of HDAC10 in tumor tissues versus normal tissues. The remaining subplots analyzed survival differences between patients with high/low HDAC10 expression groups across different cohorts (GSE9893, GSE61304, GSE42568, GSE22219, and TCGA-BRCA) using Kaplan-Meier curves, covering endpoints such as overall survival (OS), disease-free survival (DFS), relapse-free survival (RFS), and progression-free survival (PFS). The p-values from the log-rank test were also annotated. Fig. S3. Combination Efficacy of SAHA and Metformin in TNBC Cell Lines. A, The IC50 curves for SAHA (purple curve) and metformin (orange curve) were shown. The left panel displayed the percentage inhibition (%), while the right panel presented the combination index (CI) at each drug concentration. MDA-MB-231 cells were treated with SAHA, metformin, or both at the indicated concentrations. B, The IC50 curves for SAHA (purple curve) and metformin (orange curve) were shown. The left panel displayed the percentage inhibition (%), while the right panel presented the combination index (CI) at each drug concentration. Hs578T cells were treated with SAHA, metformin, or both at the indicated concentrations. Fig. S4. Colony formation and quantification o [file 12929_2025_1129_MOESM1_ESM.zip › Fig. S2.pdf]

A

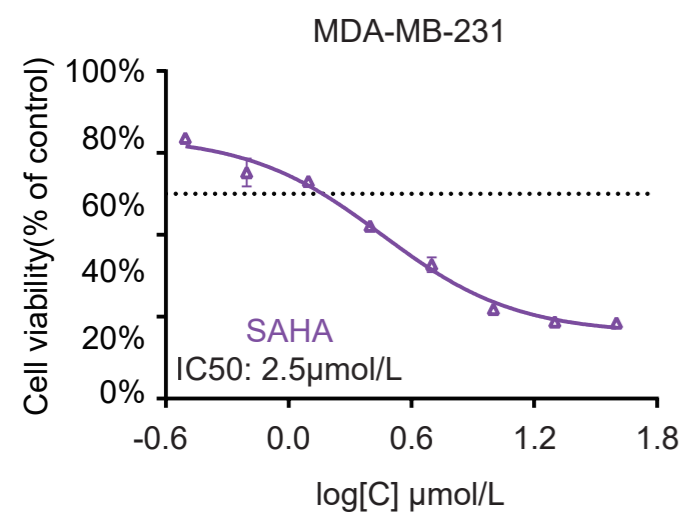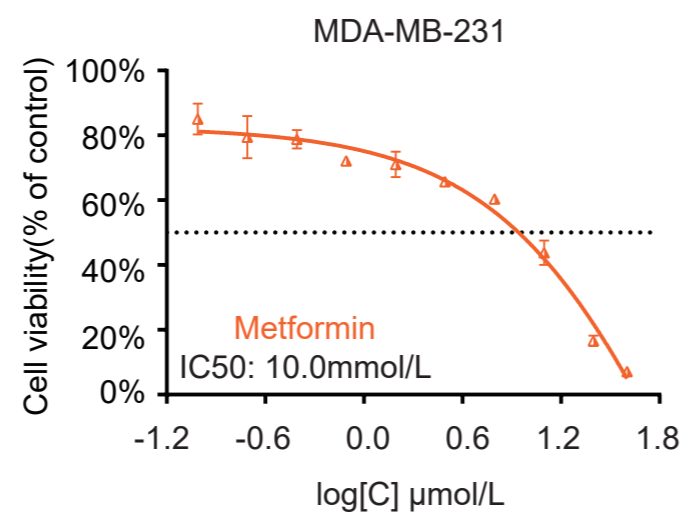

MDA-MB-231

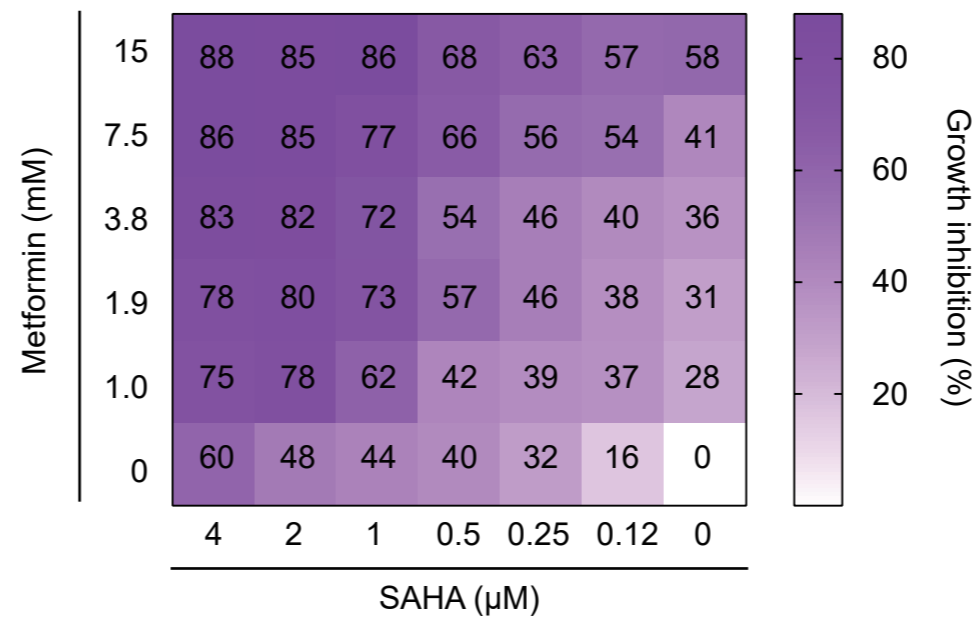

MDA-MB-231

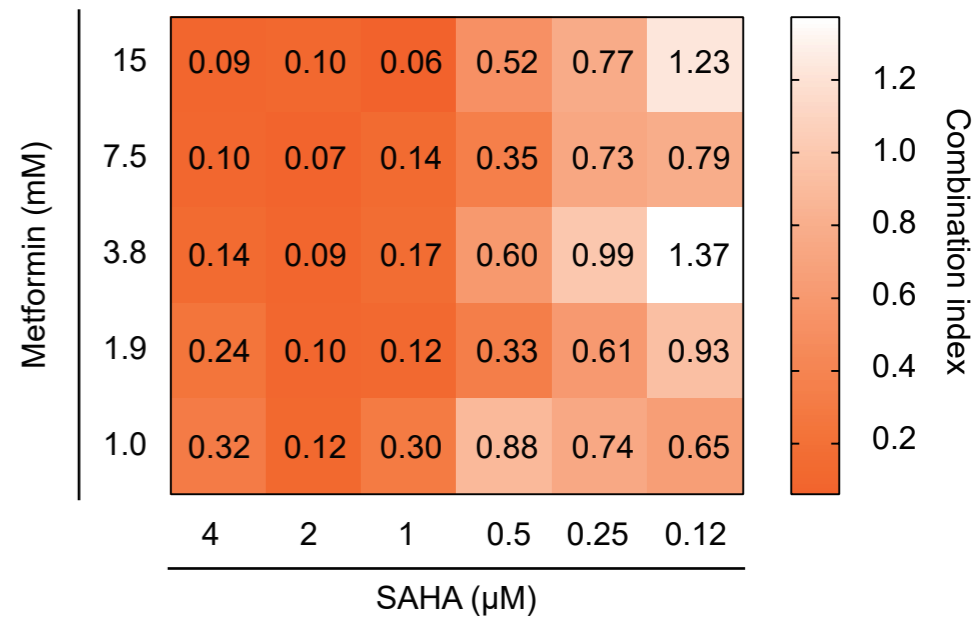

B

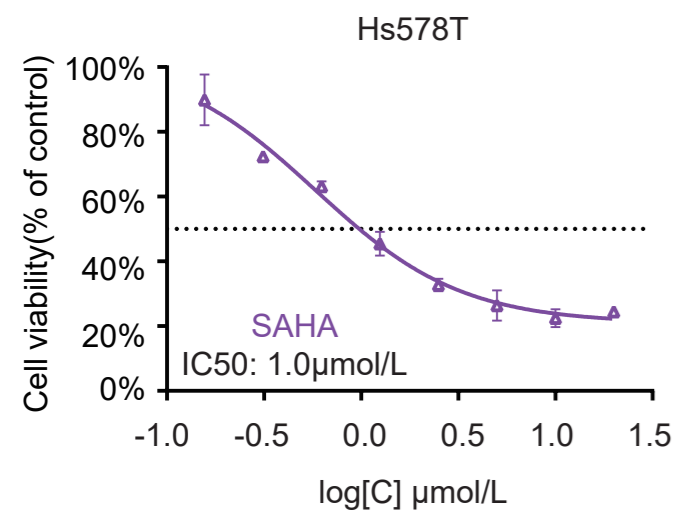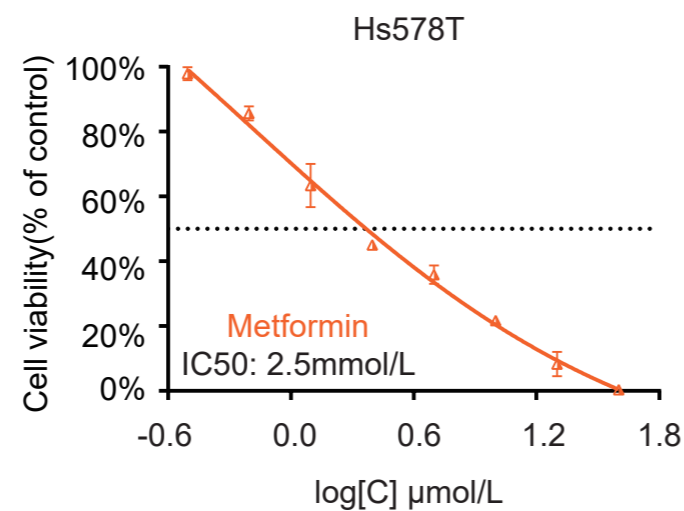

Hs578T

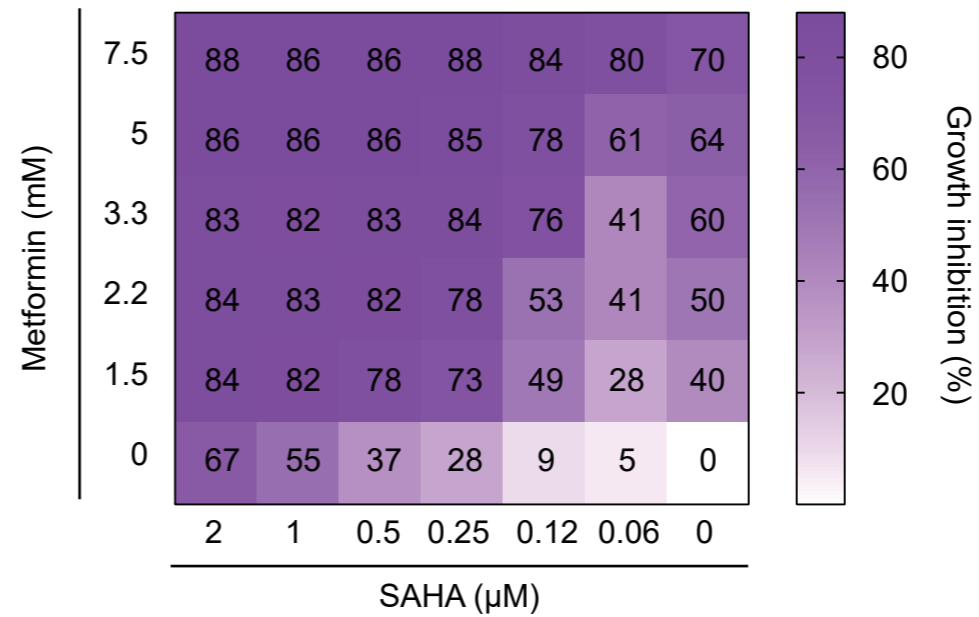

Hs578T

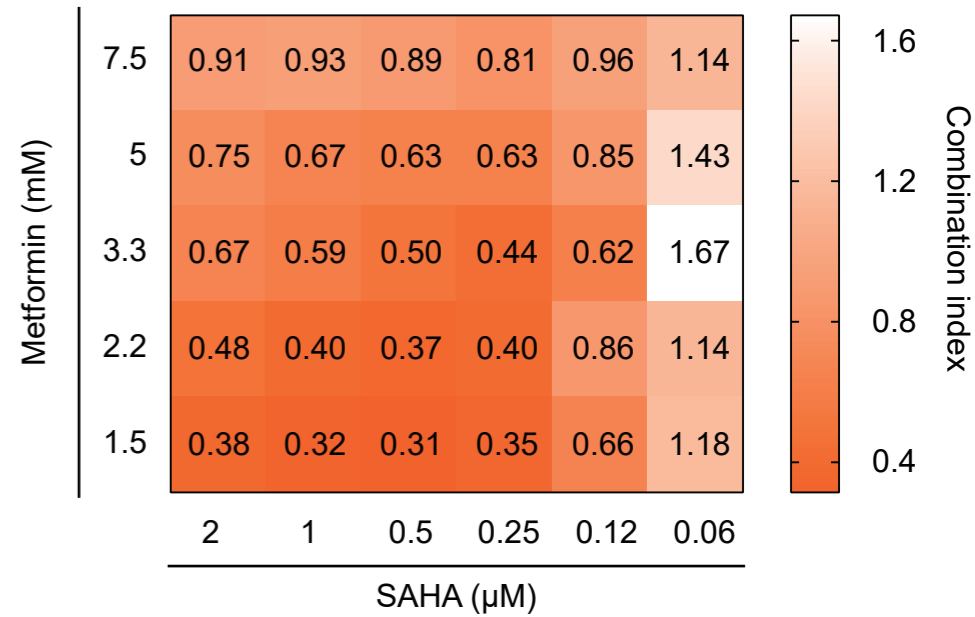

Supplement: Supplementary file 1 — Supplementary Material 1: Fig. S1. Identification of Metformin-Sensitizing Genes via CRISPR-Cas9 Screening. A, The schematic diagram showed how CDTSL identified metformin-sensitizing genes and targeted inhibitors in TNBC. B, The schematic diagram showed the composition of the CDTSL sgRNA sequences. C, The schematic diagram showed the process of CDTSL library screening. D, The Venn diagram showed how 67 candidate genes were identified through MAGeCK analysis to meet the "metformin sensitization" model. E, Functional enrichment analysis revealed a significant enrichment of histone modification-related genes. Fig. S2. The sequencing results of 1462 breast cancer patients (5 cohorts) were displayed. The scatter plot in the upper-left corner compared the expression levels of HDAC10 in tumor tissues versus normal tissues. The remaining subplots analyzed survival differences between patients with high/low HDAC10 expression groups across different cohorts (GSE9893, GSE61304, GSE42568, GSE22219, and TCGA-BRCA) using Kaplan-Meier curves, covering endpoints such as overall survival (OS), disease-free survival (DFS), relapse-free survival (RFS), and progression-free survival (PFS). The p-values from the log-rank test were also annotated. Fig. S3. Combination Efficacy of SAHA and Metformin in TNBC Cell Lines. A, The IC50 curves for SAHA (purple curve) and metformin (orange curve) were shown. The left panel displayed the percentage inhibition (%), while the right panel presented the combination index (CI) at each drug concentration. MDA-MB-231 cells were treated with SAHA, metformin, or both at the indicated concentrations. B, The IC50 curves for SAHA (purple curve) and metformin (orange curve) were shown. The left panel displayed the percentage inhibition (%), while the right panel presented the combination index (CI) at each drug concentration. Hs578T cells were treated with SAHA, metformin, or both at the indicated concentrations. Fig. S4. Colony formation and quantification o [file 12929_2025_1129_MOESM1_ESM.zip › Fig. S3.pdf]

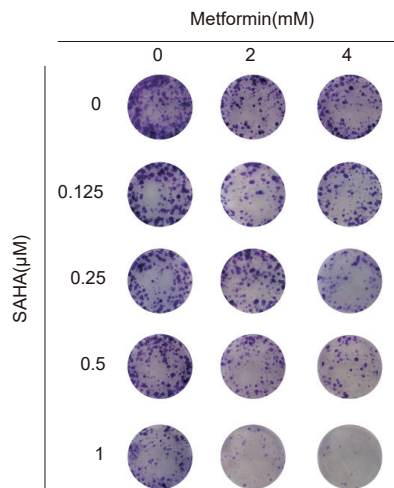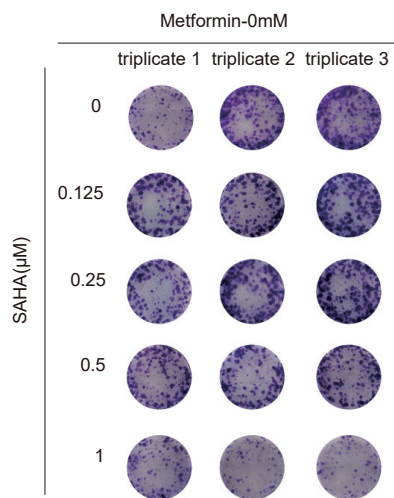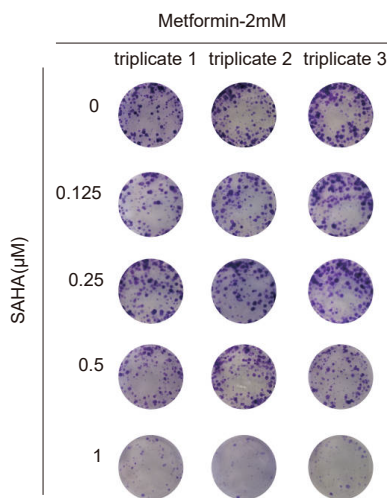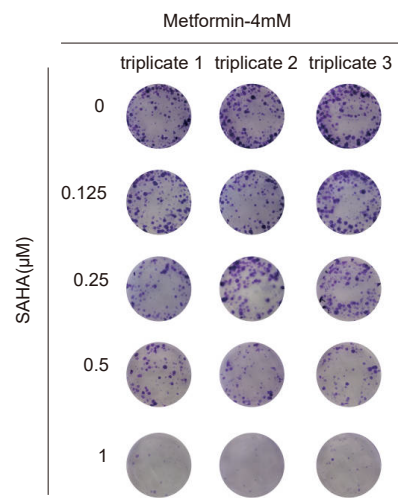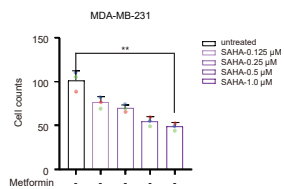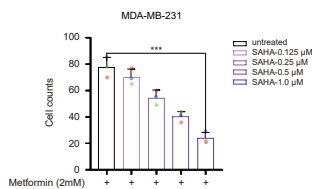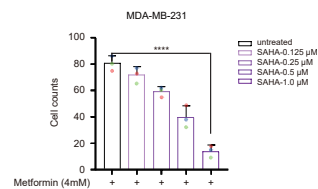

Supplement: Supplementary file 1 — Supplementary Material 1: Fig. S1. Identification of Metformin-Sensitizing Genes via CRISPR-Cas9 Screening. A, The schematic diagram showed how CDTSL identified metformin-sensitizing genes and targeted inhibitors in TNBC. B, The schematic diagram showed the composition of the CDTSL sgRNA sequences. C, The schematic diagram showed the process of CDTSL library screening. D, The Venn diagram showed how 67 candidate genes were identified through MAGeCK analysis to meet the "metformin sensitization" model. E, Functional enrichment analysis revealed a significant enrichment of histone modification-related genes. Fig. S2. The sequencing results of 1462 breast cancer patients (5 cohorts) were displayed. The scatter plot in the upper-left corner compared the expression levels of HDAC10 in tumor tissues versus normal tissues. The remaining subplots analyzed survival differences between patients with high/low HDAC10 expression groups across different cohorts (GSE9893, GSE61304, GSE42568, GSE22219, and TCGA-BRCA) using Kaplan-Meier curves, covering endpoints such as overall survival (OS), disease-free survival (DFS), relapse-free survival (RFS), and progression-free survival (PFS). The p-values from the log-rank test were also annotated. Fig. S3. Combination Efficacy of SAHA and Metformin in TNBC Cell Lines. A, The IC50 curves for SAHA (purple curve) and metformin (orange curve) were shown. The left panel displayed the percentage inhibition (%), while the right panel presented the combination index (CI) at each drug concentration. MDA-MB-231 cells were treated with SAHA, metformin, or both at the indicated concentrations. B, The IC50 curves for SAHA (purple curve) and metformin (orange curve) were shown. The left panel displayed the percentage inhibition (%), while the right panel presented the combination index (CI) at each drug concentration. Hs578T cells were treated with SAHA, metformin, or both at the indicated concentrations. Fig. S4. Colony formation and quantification o [file 12929_2025_1129_MOESM1_ESM.zip › Fig. S4.pdf]

A

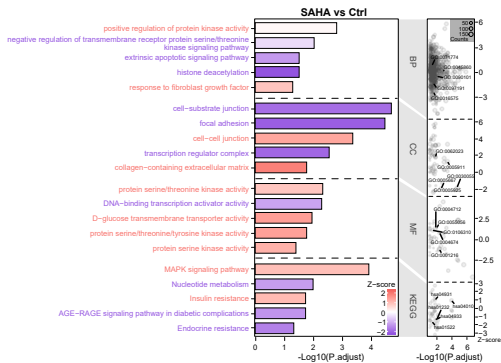

B

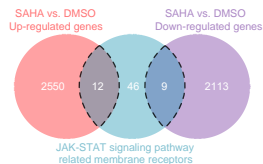

C

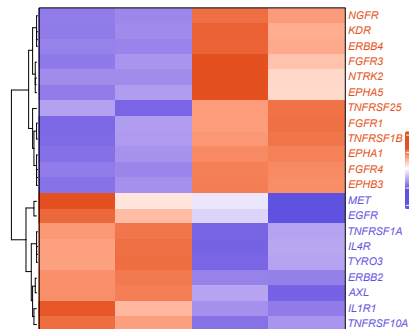

D

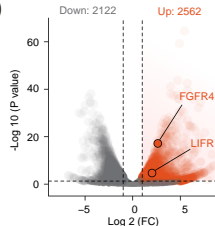

E

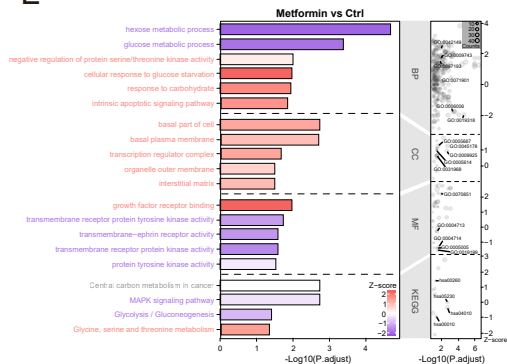

F

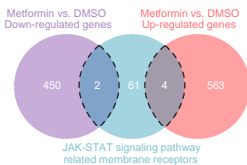

H

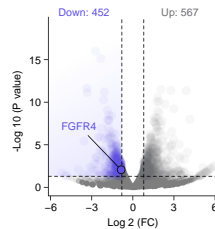

G

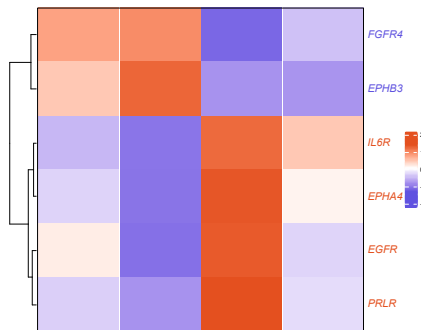

Supplement: Supplementary file 1 — Supplementary Material 1: Fig. S1. Identification of Metformin-Sensitizing Genes via CRISPR-Cas9 Screening. A, The schematic diagram showed how CDTSL identified metformin-sensitizing genes and targeted inhibitors in TNBC. B, The schematic diagram showed the composition of the CDTSL sgRNA sequences. C, The schematic diagram showed the process of CDTSL library screening. D, The Venn diagram showed how 67 candidate genes were identified through MAGeCK analysis to meet the "metformin sensitization" model. E, Functional enrichment analysis revealed a significant enrichment of histone modification-related genes. Fig. S2. The sequencing results of 1462 breast cancer patients (5 cohorts) were displayed. The scatter plot in the upper-left corner compared the expression levels of HDAC10 in tumor tissues versus normal tissues. The remaining subplots analyzed survival differences between patients with high/low HDAC10 expression groups across different cohorts (GSE9893, GSE61304, GSE42568, GSE22219, and TCGA-BRCA) using Kaplan-Meier curves, covering endpoints such as overall survival (OS), disease-free survival (DFS), relapse-free survival (RFS), and progression-free survival (PFS). The p-values from the log-rank test were also annotated. Fig. S3. Combination Efficacy of SAHA and Metformin in TNBC Cell Lines. A, The IC50 curves for SAHA (purple curve) and metformin (orange curve) were shown. The left panel displayed the percentage inhibition (%), while the right panel presented the combination index (CI) at each drug concentration. MDA-MB-231 cells were treated with SAHA, metformin, or both at the indicated concentrations. B, The IC50 curves for SAHA (purple curve) and metformin (orange curve) were shown. The left panel displayed the percentage inhibition (%), while the right panel presented the combination index (CI) at each drug concentration. Hs578T cells were treated with SAHA, metformin, or both at the indicated concentrations. Fig. S4. Colony formation and quantification o [file 12929_2025_1129_MOESM1_ESM.zip › Fig. S5.pdf]

A

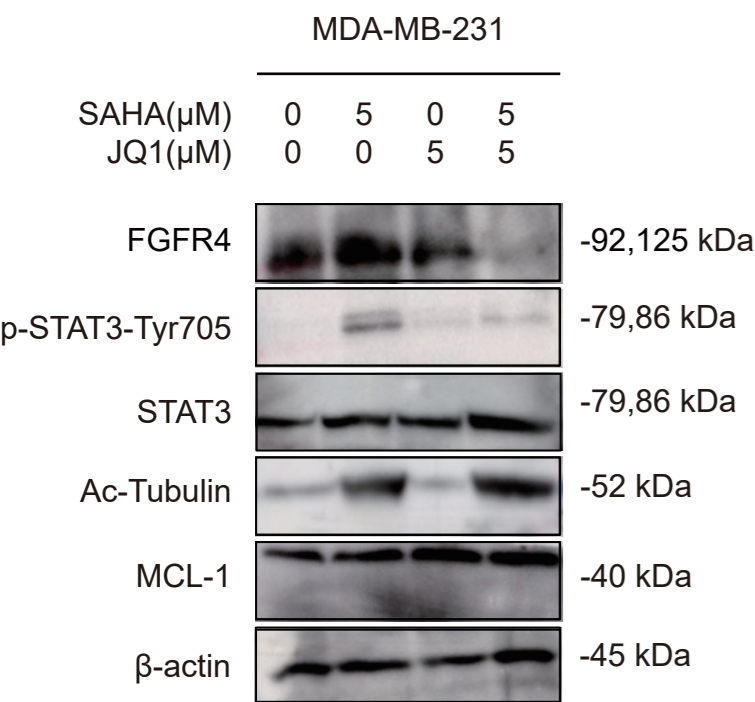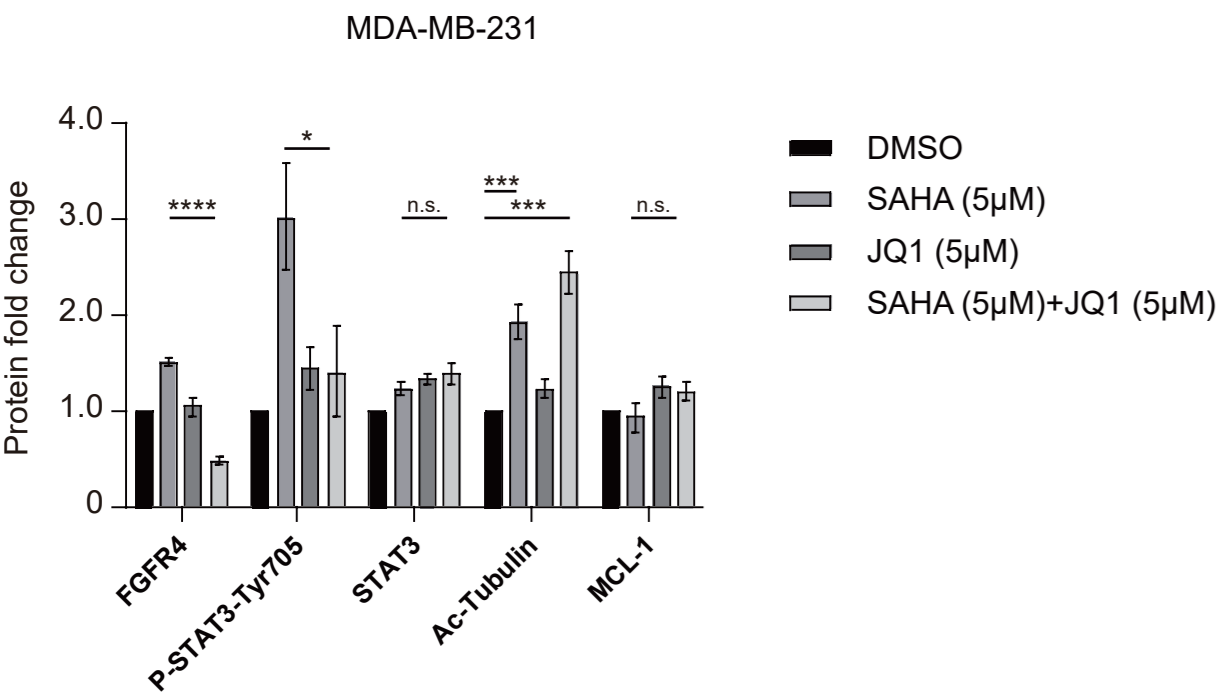

B

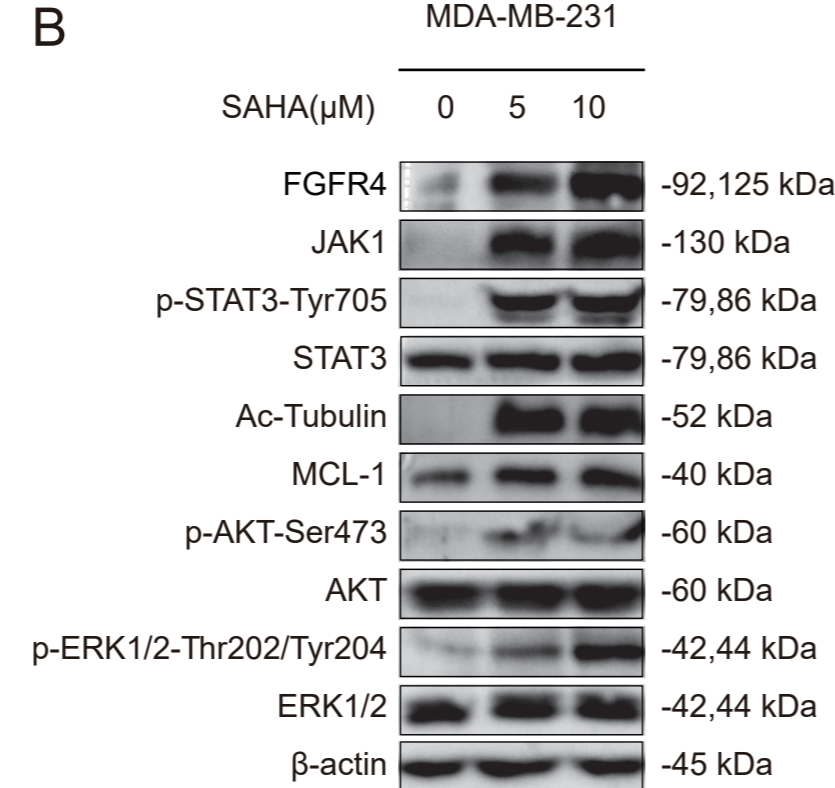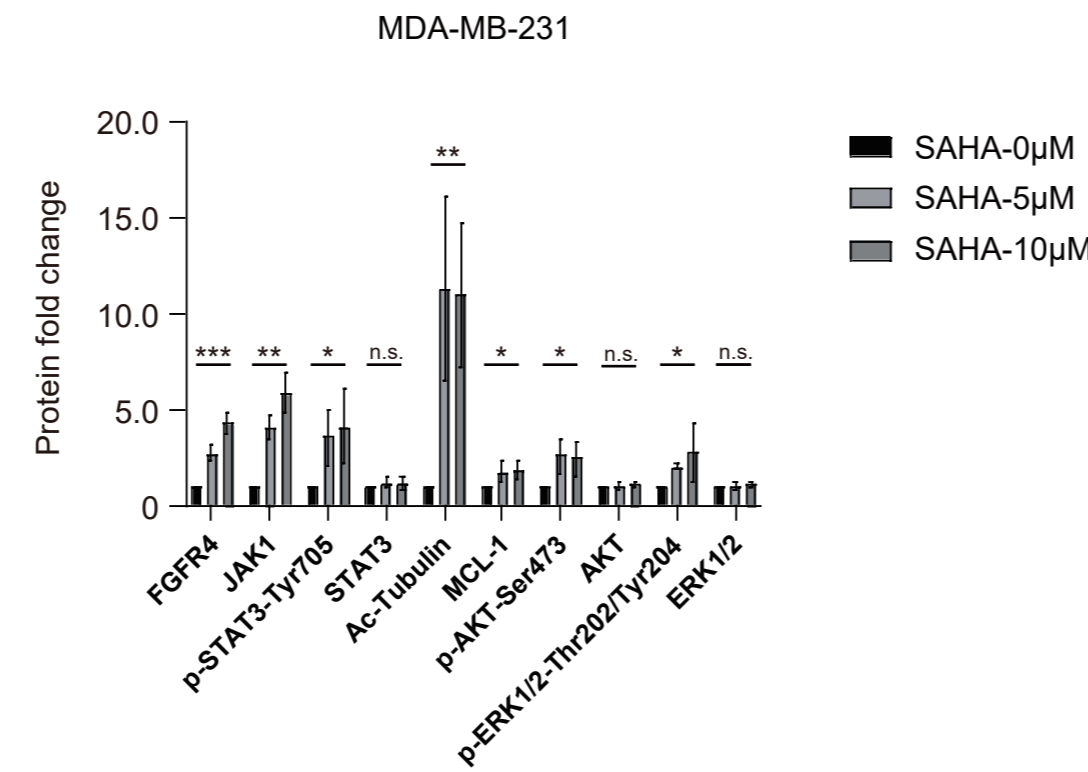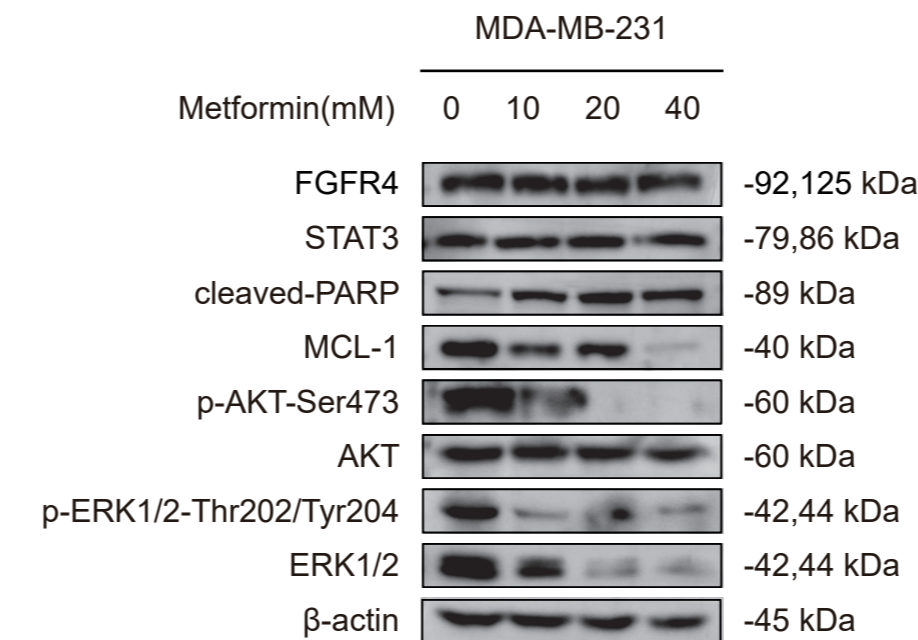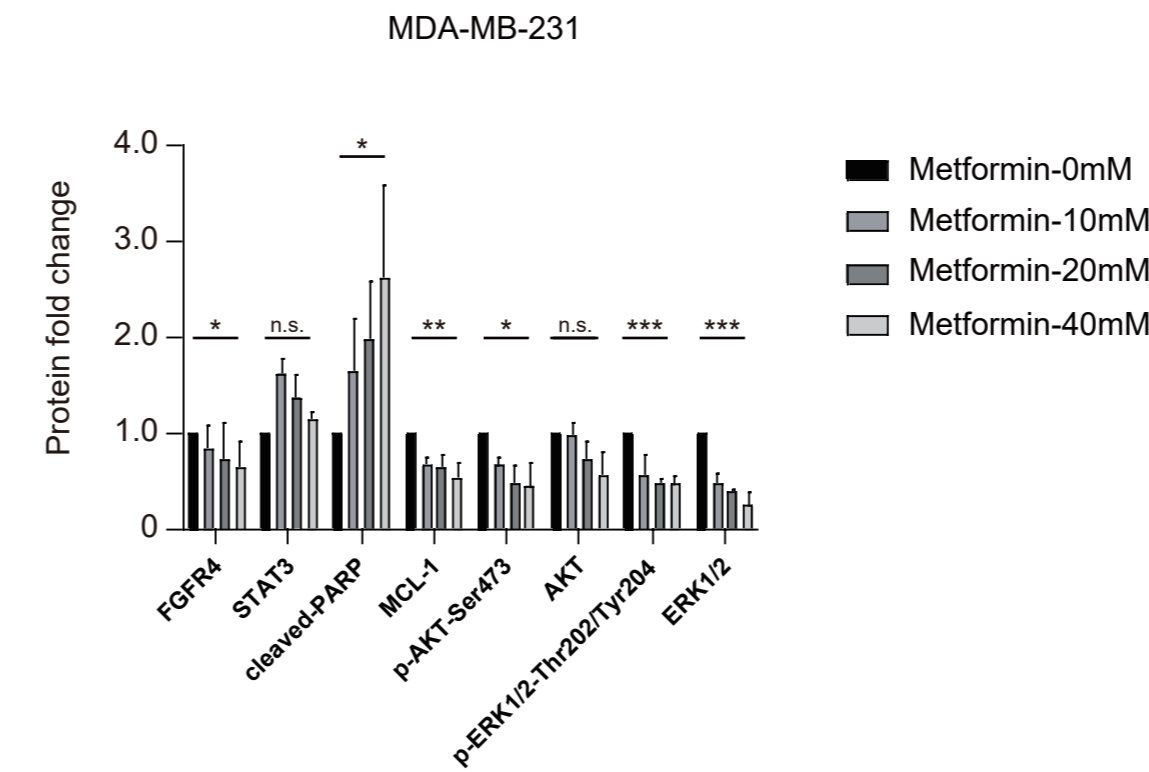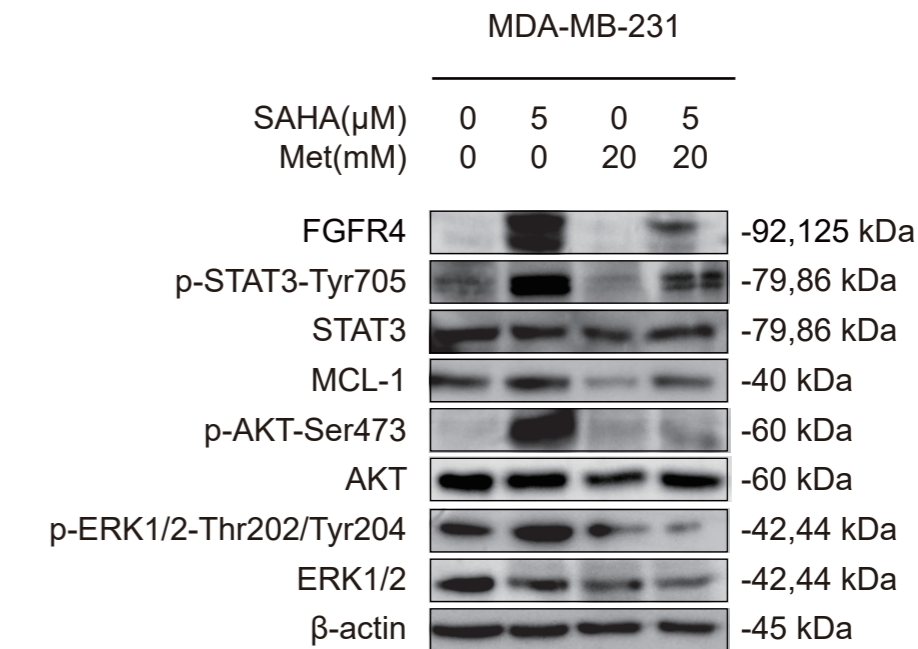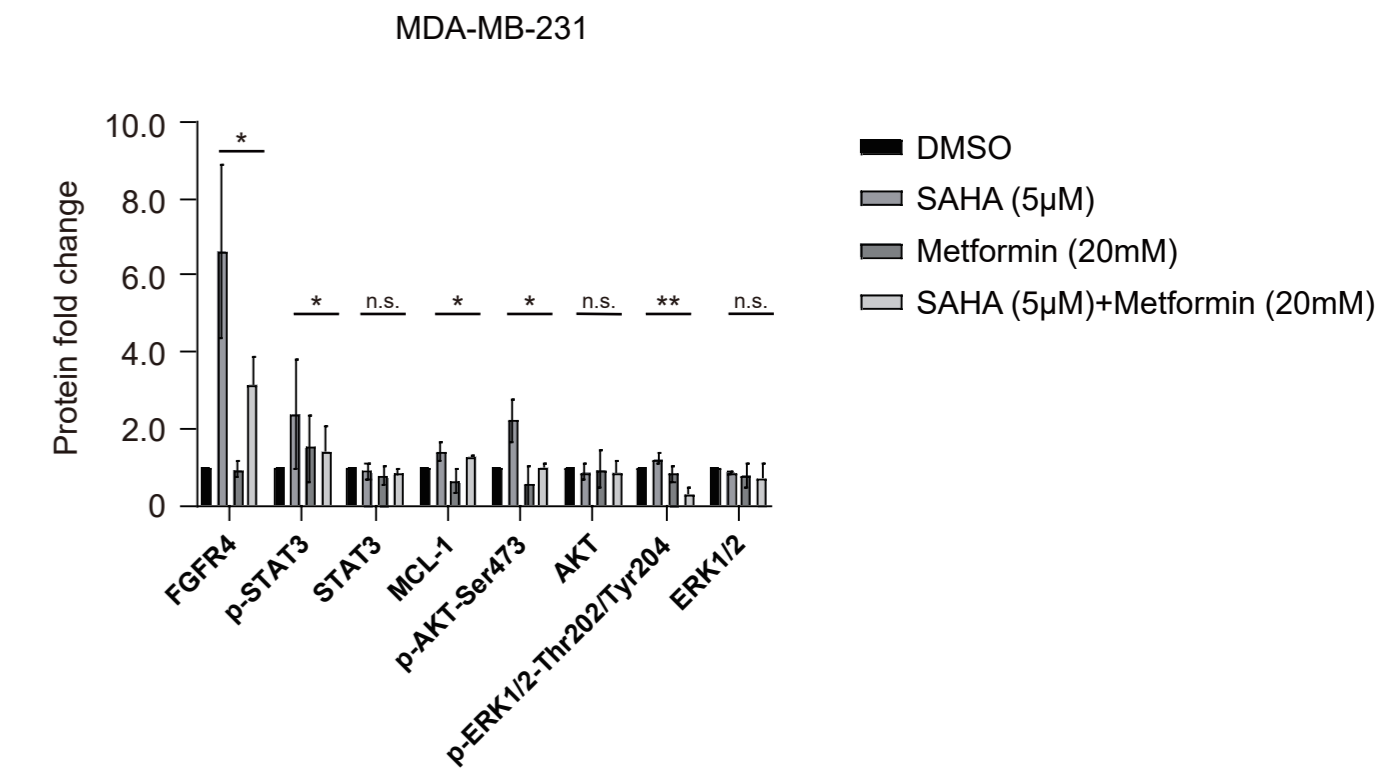

Supplement: Supplementary file 1 — Supplementary Material 1: Fig. S1. Identification of Metformin-Sensitizing Genes via CRISPR-Cas9 Screening. A, The schematic diagram showed how CDTSL identified metformin-sensitizing genes and targeted inhibitors in TNBC. B, The schematic diagram showed the composition of the CDTSL sgRNA sequences. C, The schematic diagram showed the process of CDTSL library screening. D, The Venn diagram showed how 67 candidate genes were identified through MAGeCK analysis to meet the "metformin sensitization" model. E, Functional enrichment analysis revealed a significant enrichment of histone modification-related genes. Fig. S2. The sequencing results of 1462 breast cancer patients (5 cohorts) were displayed. The scatter plot in the upper-left corner compared the expression levels of HDAC10 in tumor tissues versus normal tissues. The remaining subplots analyzed survival differences between patients with high/low HDAC10 expression groups across different cohorts (GSE9893, GSE61304, GSE42568, GSE22219, and TCGA-BRCA) using Kaplan-Meier curves, covering endpoints such as overall survival (OS), disease-free survival (DFS), relapse-free survival (RFS), and progression-free survival (PFS). The p-values from the log-rank test were also annotated. Fig. S3. Combination Efficacy of SAHA and Metformin in TNBC Cell Lines. A, The IC50 curves for SAHA (purple curve) and metformin (orange curve) were shown. The left panel displayed the percentage inhibition (%), while the right panel presented the combination index (CI) at each drug concentration. MDA-MB-231 cells were treated with SAHA, metformin, or both at the indicated concentrations. B, The IC50 curves for SAHA (purple curve) and metformin (orange curve) were shown. The left panel displayed the percentage inhibition (%), while the right panel presented the combination index (CI) at each drug concentration. Hs578T cells were treated with SAHA, metformin, or both at the indicated concentrations. Fig. S4. Colony formation and quantification o [file 12929_2025_1129_MOESM1_ESM.zip › Fig. S6.pdf]

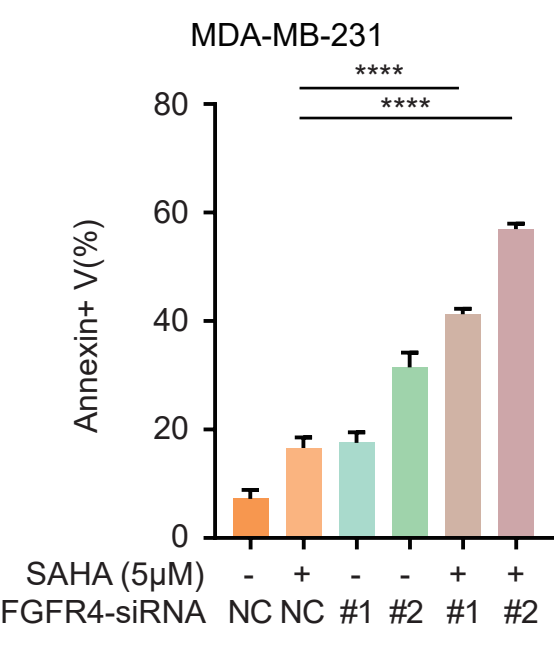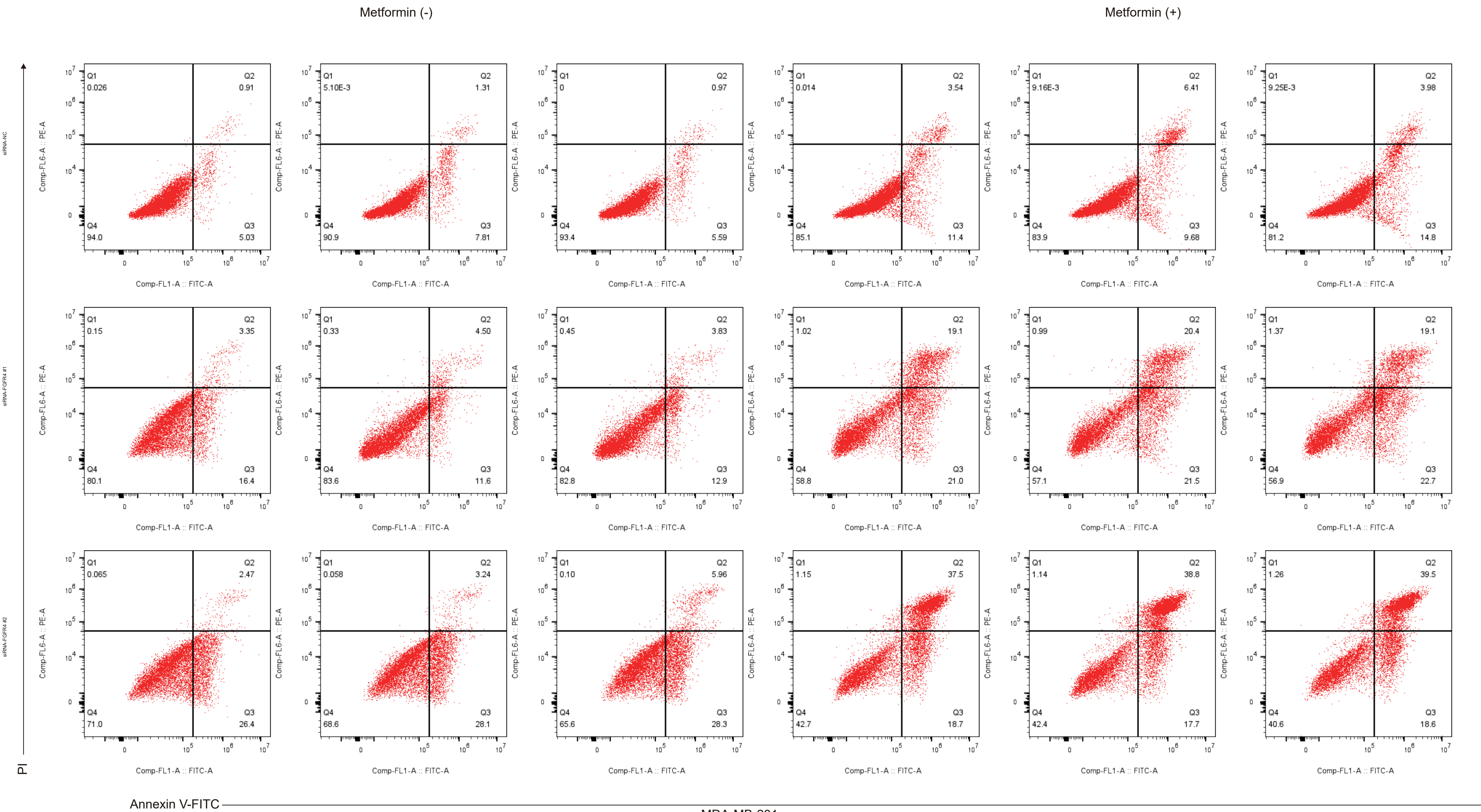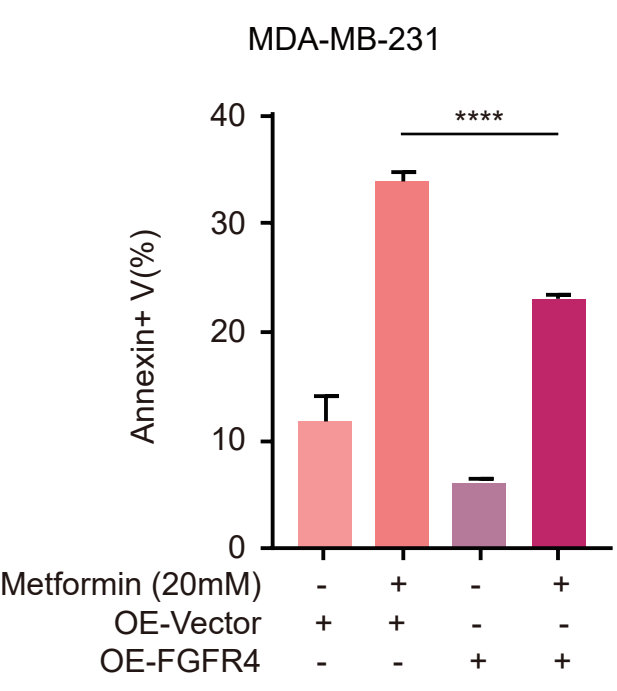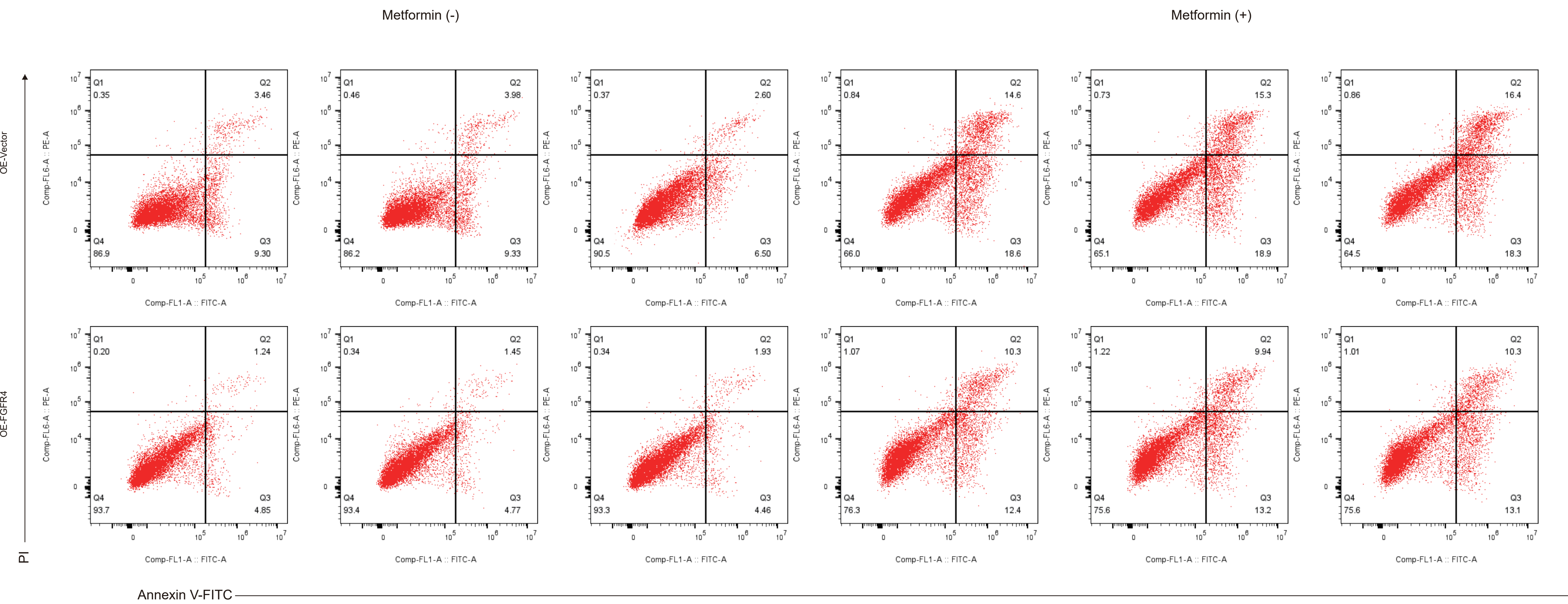

Supplement: Supplementary file 1 — Supplementary Material 1: Fig. S1. Identification of Metformin-Sensitizing Genes via CRISPR-Cas9 Screening. A, The schematic diagram showed how CDTSL identified metformin-sensitizing genes and targeted inhibitors in TNBC. B, The schematic diagram showed the composition of the CDTSL sgRNA sequences. C, The schematic diagram showed the process of CDTSL library screening. D, The Venn diagram showed how 67 candidate genes were identified through MAGeCK analysis to meet the "metformin sensitization" model. E, Functional enrichment analysis revealed a significant enrichment of histone modification-related genes. Fig. S2. The sequencing results of 1462 breast cancer patients (5 cohorts) were displayed. The scatter plot in the upper-left corner compared the expression levels of HDAC10 in tumor tissues versus normal tissues. The remaining subplots analyzed survival differences between patients with high/low HDAC10 expression groups across different cohorts (GSE9893, GSE61304, GSE42568, GSE22219, and TCGA-BRCA) using Kaplan-Meier curves, covering endpoints such as overall survival (OS), disease-free survival (DFS), relapse-free survival (RFS), and progression-free survival (PFS). The p-values from the log-rank test were also annotated. Fig. S3. Combination Efficacy of SAHA and Metformin in TNBC Cell Lines. A, The IC50 curves for SAHA (purple curve) and metformin (orange curve) were shown. The left panel displayed the percentage inhibition (%), while the right panel presented the combination index (CI) at each drug concentration. MDA-MB-231 cells were treated with SAHA, metformin, or both at the indicated concentrations. B, The IC50 curves for SAHA (purple curve) and metformin (orange curve) were shown. The left panel displayed the percentage inhibition (%), while the right panel presented the combination index (CI) at each drug concentration. Hs578T cells were treated with SAHA, metformin, or both at the indicated concentrations. Fig. S4. Colony formation and quantification o [file 12929_2025_1129_MOESM1_ESM.zip › Fig. S8.pdf]

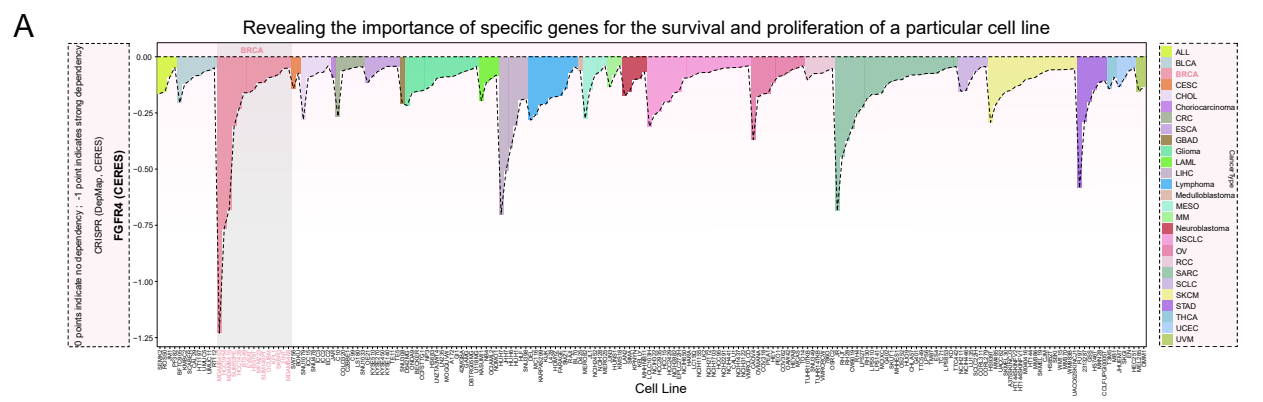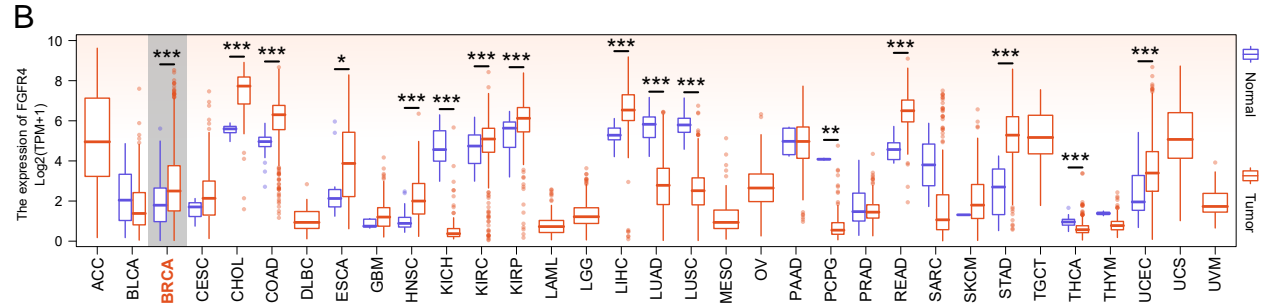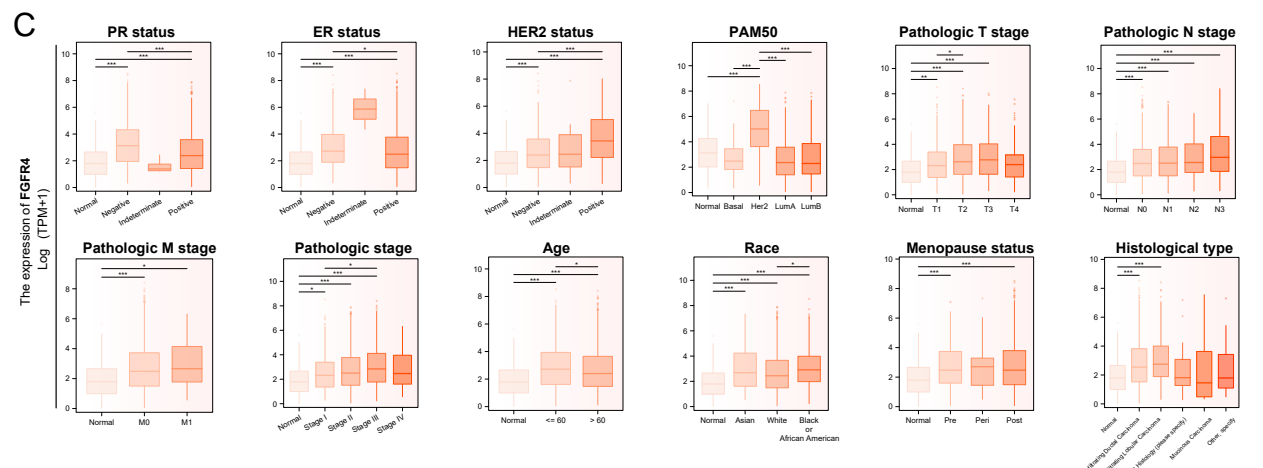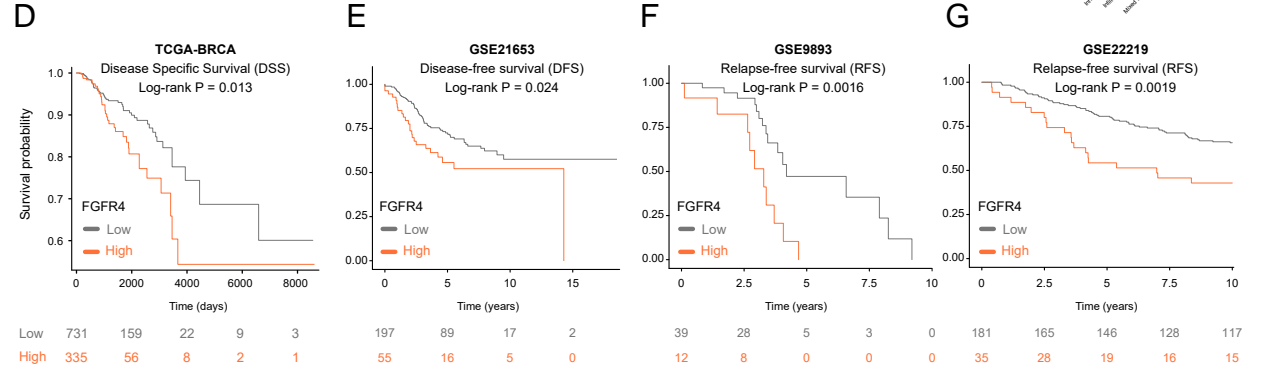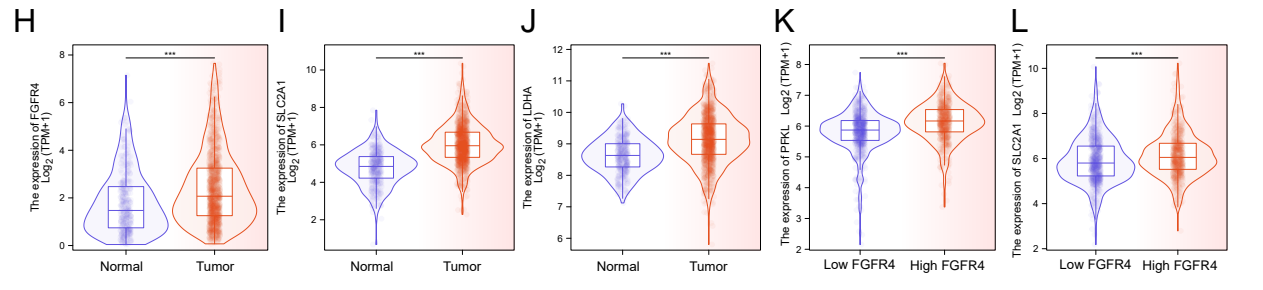

Supplement: Supplementary file 1 — Supplementary Material 1: Fig. S1. Identification of Metformin-Sensitizing Genes via CRISPR-Cas9 Screening. A, The schematic diagram showed how CDTSL identified metformin-sensitizing genes and targeted inhibitors in TNBC. B, The schematic diagram showed the composition of the CDTSL sgRNA sequences. C, The schematic diagram showed the process of CDTSL library screening. D, The Venn diagram showed how 67 candidate genes were identified through MAGeCK analysis to meet the "metformin sensitization" model. E, Functional enrichment analysis revealed a significant enrichment of histone modification-related genes. Fig. S2. The sequencing results of 1462 breast cancer patients (5 cohorts) were displayed. The scatter plot in the upper-left corner compared the expression levels of HDAC10 in tumor tissues versus normal tissues. The remaining subplots analyzed survival differences between patients with high/low HDAC10 expression groups across different cohorts (GSE9893, GSE61304, GSE42568, GSE22219, and TCGA-BRCA) using Kaplan-Meier curves, covering endpoints such as overall survival (OS), disease-free survival (DFS), relapse-free survival (RFS), and progression-free survival (PFS). The p-values from the log-rank test were also annotated. Fig. S3. Combination Efficacy of SAHA and Metformin in TNBC Cell Lines. A, The IC50 curves for SAHA (purple curve) and metformin (orange curve) were shown. The left panel displayed the percentage inhibition (%), while the right panel presented the combination index (CI) at each drug concentration. MDA-MB-231 cells were treated with SAHA, metformin, or both at the indicated concentrations. B, The IC50 curves for SAHA (purple curve) and metformin (orange curve) were shown. The left panel displayed the percentage inhibition (%), while the right panel presented the combination index (CI) at each drug concentration. Hs578T cells were treated with SAHA, metformin, or both at the indicated concentrations. Fig. S4. Colony formation and quantification o [file 12929_2025_1129_MOESM1_ESM.zip › Fig. S9.pdf]
